# Paediatric Scalp-Hair Biomonitoring of Nutritionally Relevant and Mixed-Source Elements in Urban–Industrial Spain

**DOI:** 10.3390/jox16050157

**Published:** 2026-08-25

**Authors:** Antonio Peña-Fernández, M. Ángeles Peña Fernández, Borja Martínez-Alonso, Rafael Moreno-Gómez-Toledano, Tomás Cámara-Pastor, Manuel Higueras, M. Carmen Lobo-Bedmar

**Affiliations:** 1 Area of Legal and Forensic Medicine, Department of Surgery, Medical and Social Sciences, Faculty of Medicine and Health Sciences, University of Alcalá, Crta. Madrid-Barcelona Km, 33.6, 28871 Alcalá de Henares, Spain; 2 Department of Biomedical Sciences, Faculty of Pharmacy, University of Alcalá, Crta. Madrid-Barcelona Km, 33.6, 28871 Alcalá de Henares, Spain; 3 Area of Human Anatomy and Embryology, Faculty of Medicine and Health Sciences, University of Alcalá, Crta. Madrid-Barcelona Km, 33.6, 28871 Alcalá de Henares, Spain; 4 Scientific Computation Research Institute (SCRIUR), University of La Rioja, 26006 Logroño, Spain; 5 Departamento de Investigación Agroambiental, Madrid Institute for Rural, Agricultural and Food Research and Development (IMIDRA), Finca el Encín, Crta. Madrid-Barcelona Km, 38.2, 28800 Alcalá de Henares, Spain

**Keywords:** scalp hair, paediatric biomonitoring, children, adolescents, elemental biomonitoring, calcium, magnesium, nickel, left-censored data, urban–industrial environment

## Abstract

Scalp hair offers a non-invasive matrix for retrospective paediatric biomonitoring, although interpretation is complicated by endogenous incorporation, exogenous deposition and element-specific analytical detectability. We characterised B, Ca, Co, Fe, Li, Mg, Mo, Ni and Se in archived scalp hair collected in 2001 from 120 children aged 6–9 years and 97 adolescents aged 13–16 years residing in urban–industrial Alcalá de Henares, Spain. Samples were analysed in 2025 by ICP–MS using a common quality-controlled analytical workflow, with left-censored observations evaluated using censoring-aware methods. B and Li were highly censored (89.2–94.8% and 86.7–99.0%, respectively), whereas Fe, Mg, Mo and Se were consistently quantified. Adolescents showed higher median Ca (325 vs. 168 µg g^−1^; *q* = 1.43 × 10^−9^), Mg (24.0 vs. 9.05 µg g^−1^; *q* = 8.91 × 10^−19^) and Ni (0.169 vs. 0.0597 µg g^−1^; *q* = 2.10 × 10^−5^), but lower Fe (5.16 vs. 7.54 µg g^−1^; *q* = 2.28 × 10^−11^), Co and Mo than children. Sex-associated differences were also observed for several elements, although their magnitude and detectability varied between age strata. Residential heterogeneity was strongest among adolescents: median Ca in the mixed residential–industrial Zone IV was 967 µg g^−1^ compared with 232–385 µg g^−1^ in Zones I–III (*q* = 2.75 × 10^−12^), while median Mg was 113 versus 18.8–24.3 µg g^−1^ (*q* = 2.26 × 10^−6^). These specimens, collected in 2001, stored dry in paper envelopes for approximately 24 years, and analysed together in the 2025 ICP–MS campaign, provide a distinctive historical paediatric biomonitoring baseline from a well-defined Spanish urban–industrial population. The findings support scalp hair as a screening-level matrix for investigating population patterns across developmental stage, sex and residential setting, while not supporting its use as a direct measure of nutritional status, internal dose or health risk.

## 1. Introduction

Urban–industrial environments generate complex mixtures of elements through the combined influence of local geology, legacy deposition, industrial activities, traffic-related abrasion, construction materials, and resuspended soil and dust [1]. These mixtures are particularly relevant in paediatric populations because contact with outdoor soil and indoor dust varies with age, behaviour, mobility, and residential setting [2]. Recent exposure-science research has refined age-specific estimates of soil and dust ingestion and the characterisation of activity-related exposure pathways [3,4,5]. However, interpretation of paediatric biomonitoring remains challenging when measured elements may reflect physiological regulation, environmental contribution, or both.

The elements considered in the present study occupy different positions along a homeostasis-to-exposure continuum. Calcium (Ca) and magnesium (Mg) are major minerals involved in skeletal development and neuromuscular function, with internal concentrations primarily governed by dietary intake, absorption, and endocrine regulation [6,7]. Iron (Fe) is essential for oxygen transport, neurodevelopment, and numerous enzymatic processes [8]. Selenium (Se) contributes to antioxidant defence, immune function, and thyroid metabolism, whereas molybdenum (Mo) is required for enzymes involved in sulfur and purine metabolism [9,10]. Cobalt (Co) is nutritionally relevant through its presence in vitamin B12, although environmental Co may also reflect geological and anthropogenic inputs [11]. Nickel (Ni) is more appropriately considered an exposure-relevant element because of its environmental occurrence and recognised importance in sensitised individuals [12]. Boron (B) and lithium (Li) have multiple potential determinants, including diet, drinking water, environmental media, and consumer products, and consequently require cautious source interpretation [13,14,15].

Human biomonitoring provides an integrated approach to characterising exposure, but comparability depends on harmonised analytical procedures and biomatrix-specific interpretation [16]. Scalp hair is particularly attractive for paediatric studies because collection is non-invasive, storage is straightforward, and archived specimens permit retrospective population-level investigation. Nevertheless, hair-element concentrations may reflect endogenous incorporation during growth, exogenous deposition on the hair surface, or a combination of both. They may also be influenced by hair characteristics, washing and digestion procedures, analytical sensitivity, and the treatment of observations below the limit of detection. Moreover, harmonised paediatric reference distributions for hair remain less established than those available for conventional biomonitoring matrices [17]. Hair should therefore be interpreted as a screening matrix rather than as a direct measure of nutritional adequacy, internal dose, or health risk.

Available Spanish studies demonstrate the value of paediatric hair-element profiling while also illustrating important limitations in comparability and interpretation. A study conducted in the Madrid region reported reference distributions for several trace elements in children and adolescents, including Co, Fe, Mo, Ni, and Se [18]. A subsequent study in eastern Spain quantified 28 elements in children’s hair by ICP–MS and proposed provisional reference values for several analytes overlapping with the present panel [19]. These studies provide important national comparators, but differences in population structure, analytical procedures, sensitivity, and treatment of non-detects complicate direct comparison. The archived Alcalá cohort offers a complementary opportunity because children and adolescents from the same urban–industrial municipality can be evaluated within a common contemporary analytical and censoring-aware framework. Its value therefore lies not simply in generating additional hair concentration data, but in providing a harmonised historical population profile across two developmental stages and contrasting residential settings that may serve as a baseline for future temporal and population comparisons.

Environmental information may provide useful ecological context for biomonitoring findings without establishing individual-level source attribution. The GEMAS project provides harmonised information on European soil geochemistry, including regional interpretation for the Iberian Peninsula [20,21], while co-temporal topsoil data from Alcalá de Henares provide a local environmental reference for overlapping analytes [22]. In the present study, these regional and local soil data are used only as secondary ecological context and a constraint on environmental plausibility; they are not treated as participant-level exposure surrogates or as evidence of direct soil-to-hair transfer.

Against this background, the present study characterises B, Ca, Co, Fe, Li, Mg, Mo, Ni, and Se in archived scalp hair collected in 2001 from children aged 6–9 years and adolescents aged 13–16 years residing in Alcalá de Henares, an urban–industrial municipality in central Spain, and analysed within a common ICP–MS campaign in 2025. The study was designed to provide a historical, population-level biomonitoring profile rather than to assess individual nutritional status, clinical health, internal dose, or source-specific exposure. Specifically, the objectives were to: (1) characterise element distributions, analytical detectability, and left-censoring; (2) evaluate developmental-stage and sex-related differences; (3) examine residential/environmental heterogeneity within each age stratum; and (4) interpret the principal findings against relevant Spanish paediatric data and secondary environmental context. By combining a well-defined historical cohort, two developmental stages, common analytical processing, and censoring-aware statistical treatment, the study aims to provide a robust historical comparator for future paediatric biomonitoring while distinguishing biologically and environmentally plausible population patterns from signals that remain limited by matrix characteristics or analytical censoring.

## 2. Materials and Methods

### 2.1. Study Area and Design

Alcalá de Henares (40°29′ N, 3°22′ W) is a medium-sized city in central Spain located approximately 30 km east of Madrid. The municipality comprises compact residential districts, lower-density neighbourhoods, major traffic corridors, green areas, and mixed residential–industrial settings, providing a heterogeneous urban framework for investigating population-level variation in paediatric hair-element profiles.

The present retrospective biomonitoring study used archived scalp-hair specimens collected in Alcalá de Henares in 2001. All available specimens included in the present analysis were retrieved from storage and processed in 2025 within a single analytical campaign. The same hair-decontamination, digestion, instrumental, and analytical quality-assurance and quality-control workflow was applied to both age strata, ensuring internal comparability between children and adolescents and across residential zones [23,24].

### 2.2. Participants, Ethics, and Inclusion Criteria

The study comprised 217 participants divided into two paediatric age strata: 120 children aged 6–9 years, including 50 boys and 70 girls, and 97 adolescents aged 13–16 years, including 29 males and 68 females.

Eligibility criteria were established during the original recruitment to minimise major non-exposure sources of variability in hair elemental composition. Participants had been born in Alcalá de Henares and had lived in the municipality throughout their lives, had naturally dark and untreated hair, reported no use of hair dyes or chemical straightening treatments, and were not taking regular medication at the time of sampling. Such restrictions are commonly applied in hair-element biomonitoring to reduce variation associated with cosmetic treatments, hair characteristics, and external contamination [25,26].

Sampling was conducted during April–May 2001 as part of a municipal biomonitoring programme designed to characterise baseline exposure patterns in the paediatric population. The investigation complied with the principles of the Declaration of Helsinki. Written informed consent was obtained from parents or legal guardians, and assent was obtained from participants where appropriate. The retrospective analytical study and its reporting framework were approved by the University of Alcalá Research Ethics Committee (Comité de Ética de la Investigación y de Experimentación Animal, CEI-EA; protocol code CEIP/2025/3/089; approval date: 5 May 2025).

### 2.3. Hair Sampling, Handling, and Residential Zoning

Scalp hair was collected from the occipital region and cut as close as possible to the scalp using stainless-steel scissors. During the original 2001 sampling, the proximal hair fraction was retained for analysis. For participants with longer hair, approximately the first 2–3 cm from the scalp were retained, and the remaining distal portion was discarded. This procedure was intended to focus the analysis on the most recent period of hair growth and to improve comparability with younger children, who generally had shorter hair.

Samples were handled using powder-free gloves and stored dry in clean paper envelopes until laboratory processing. The archived material analysed in 2025 therefore corresponded to the proximal hair fractions selected during the original sampling. No additional retrospective segmentation was performed, and the samples were analysed as proximal bulk-hair specimens rather than as sequential hair segments. The results consequently represent concentrations in an approximate recent retrospective growth window rather than segment-specific temporal profiles. The exact period represented remains uncertain because of inter-individual variation in hair growth rate and in the precise length of the available proximal fraction [26,27].

For spatial interpretation, Alcalá de Henares was divided a priori into four residential/environmental zones using the framework applied in previous studies of the same cohort [23,24] and illustrated in Supplementary Figure S1. Zone I corresponded to lower-density residential areas with greater green-space availability; Zone II represented the compact urban and residential core; Zone III comprised areas dominated by major road traffic; and Zone IV represented the most heterogeneous land-use setting, combining residential areas with industrial land use and proximity to mapped potential pollution-source locations. This zoning was used as an ecological residential-setting classification; the historical dataset did not contain a sufficiently detailed participant-linked inventory of individual industrial facilities or activities to support attribution to specific industrial processes or emission sources.

Differences in zone coverage between age strata reflected the original school-based recruitment frame and the availability of archived specimens rather than post hoc exclusion. Children were recruited from schools in Zones I, II, and IV, whereas adolescents were represented across all four zones. Residential-zone analyses were therefore performed separately within each age stratum. Zone-related findings were interpreted as intra-stratum spatial contrasts and not as directly comparable child–adolescent zone effects.

### 2.4. Food-Group Consumption Frequency

Food-group consumption-frequency data collected in 2001 were retained exclusively as a descriptive context for the original study population. Habitual diet had been assessed using a brief food-frequency questionnaire covering 14 major food groups and consistent with Spanish paediatric dietary-research approaches [28,29,30,31]. The available data consisted of aggregated age- and sex-stratified frequency distributions rather than participant-linked dietary records; consequently, they were not used to estimate nutrient or element intake, assess dietary adequacy, or model diet as an individual-level determinant of hair concentrations. The complete questionnaire structure, category regrouping, valid denominators, statistical treatment, and updated descriptive results are provided in Supplementary Methods S1, Supplementary Tables S1 and S2, and Supplementary Figure S2. Where used in the present study, FFQ information was interpreted only as broad dietary background and not as evidence of a specific diet–hair pathway [32].

### 2.5. Hair Decontamination, Digestion, and Elemental Determination

Because scalp hair may retain exogenous particulate material, all specimens underwent a standardised decontamination procedure before digestion [33]. Each bulk-hair specimen, initially comprising approximately 1 g where sufficient material was available, was immersed in a 1% (*v*/*v*) Triton X-100 solution (Sigma-Aldrich, St Louis, MO, USA) prepared with ultrapure water from a Milli-Q^®^ Direct 8 purification system (Merck Millipore, Darmstadt, Germany; resistivity 18.2 MΩ cm).

Samples were sonicated for 5 min in an ultrasonic bath (J.P. Selecta Ultrasons, Abrera, Barcelona, Spain), and the detergent-washing procedure was repeated four times. Samples were subsequently rinsed three times with ultrapure water and sonicated for a further 5 min in ultrapure water alone to remove residual surfactant. Cleaned hair was placed on acid-cleaned filter paper and dried at 50 °C to constant weight.

For acid digestion, approximately 100 mg of washed and dried hair was weighed into acid-washed polytetrafluoroethylene digestion tubes and treated with 2 mL of 65% HNO_3_ (Suprapur^®^, Merck, Darmstadt, Germany). Samples were allowed to pre-oxidise for 12 h at room temperature and were subsequently heated at 96 °C for 12 h using a programmable digestion block. After cooling, each digest was quantitatively diluted to 10 mL with ultrapure water, transferred to nitric-acid-washed polypropylene tubes, and stored at −80 °C until instrumental analysis. Frozen digests were thawed overnight at room temperature before analysis.

Procedural digestion blanks were included at a frequency of approximately one blank for every five samples. These blanks were used to monitor contamination introduced during sample handling and digestion and to support calculation of the analytical limits of detection.

Boron (B), calcium (Ca), cobalt (Co), iron (Fe), lithium (Li), magnesium (Mg), molybdenum (Mo), nickel (Ni), and selenium (Se) were quantified by inductively coupled plasma–mass spectrometry using a NexION 350D instrument (PerkinElmer, Waltham, MA, USA). Measurements were performed under collision-cell conditions suitable for multi-element analysis of a keratinised matrix.

Before instrumental determination, digest solutions were diluted 1:5 with ultrapure water acidified to 1% HNO_3_ (*v*/*v*). External calibration was performed using a matrix-matched blank and multi-point standards prepared from certified TraceCERT^®^ solutions (Merck, Darmstadt, Germany) in the same acidic medium. Niobium was introduced continuously as the internal standard to correct for instrumental drift and matrix-related signal variability throughout the analytical sequence.

Each digest was analysed in duplicate, with three replicate readings per sample and an integration time of 1 s per monitored isotope. The isotopes used were ^11^B, ^44^Ca, ^59^Co, ^56^Fe, ^7^Li, ^24^Mg, ^97^Mo, ^60^Ni, and ^77^Se.

Analytical QA/QC included procedural blanks, calibration-verification standards, duplicate analyses, internal-standard monitoring, and analysis of certified reference material. NCS DC 73347 human-hair certified reference material (National Research Centre for Certified Reference Materials, Beijing, China) was used as the principal keratin-matrix control and was analysed approximately every five samples, consistent with the analytical strategy applied in previous studies of this cohort [23,24]. The concentrations obtained for NCS DC 73347 were 1.83 µg g^−1^ for Li, 1.41 µg g^−1^ for B, 2.80 × 10^3^ µg g^−1^ for Ca, 0.0655 µg g^−1^ for Co, 55.7 µg g^−1^ for Fe, 350 µg g^−1^ for Mg, 0.0680 µg g^−1^ for Mo, 0.818 µg g^−1^ for Ni, and 0.556 µg g^−1^ for Se. Based on the corresponding certified or reference concentrations, analytical recoveries were 91.3% for Li, 109% for B, 96.6% for Ca, 92.2% for Co, 103% for Fe, 97.2% for Mg, 93.2% for Mo, 98.6% for Ni, and 92.6% for Se. For B, recovery was calculated against the manufacturer-reported reference concentration of 1.30 µg g^−1^, which is provided as an uncertified reference value. The certified or reference concentrations applicable to the present elemental panel and the corresponding element-specific QA/QC information are provided in Supplementary Methods S2.

Procedural blanks and calibration-verification standards were measured at the beginning and end of each analytical sequence. Element-specific limits of detection (LoDs) were calculated as three times the standard deviation of the procedural-blank signal (3*σ*). LoDs were calculated separately for children and adolescents; minor differences between age strata reflected differences in available hair mass after washing and proximal trimming and the associated final dilution requirements rather than changes in instrumental performance.

### 2.6. Statistical Analysis and Screening-Level Contextualisation

#### 2.6.1. Left-Censored Data and Descriptive Statistics

Analytical observations reported below the element-specific limit of detection (LoD) were treated explicitly as left-censored observations and were not replaced by fixed substitution values such as zero, LoD/2, or LoD/√2. The number and percentage of censored observations were calculated for each element overall and after stratification by developmental stage, sex, and residential zone; a consolidated summary is provided in Supplementary Table S3. Statistical treatment was selected according to the degree of censoring and the amount of detectable information available, following established recommendations for environmental and biomonitoring datasets containing nondetects [34,35,36,37,38].

Uncensored variables were described using empirical statistics. For variables with low or moderate censoring and sufficient detectable information, censoring-aware distributional methods, including reverse Kaplan–Meier estimation and, where required, regression on order statistics (ROS), were used to estimate descriptive distributional parameters. When censoring was too extensive or the number of detections was insufficient to support stable estimation, results were characterised principally by sample size, detection frequency, percentage censored, analytical LoD, and detectable-range information rather than by model-dependent central estimates. Detailed implementation procedures and criteria governing distributional estimability are provided in Supplementary Methods S3.

Where supported by the available distributional information, upper-tail descriptors included the 95th percentile (P95), with corresponding confidence intervals, and the 97.5th percentile (P97.5). These values were used exclusively as population-level screening descriptors and were not interpreted as clinical, nutritional, or toxicological thresholds. Full percentile interpretation was avoided for highly censored analytes, particularly B and Li.

For child Ni, all primary analyses used the final harmonised censoring-aware analytical field derived directly from the analytical source data. The historical substitution-based field (Ni†) was retained solely for methodological transparency and was excluded from primary descriptive statistics and inference. A numerical comparison of the two representations is provided in Supplementary Table S4, while the complete record-linkage, harmonisation, and audit procedure is described in Supplementary Methods S4 [34,35,36,39,40].

#### 2.6.2. Sex, Age-Group, and Residential-Zone Comparisons

Sex-related comparisons were conducted separately within children and adolescents, age-group comparisons contrasted children with adolescents, and residential-zone analyses were conducted separately within each age stratum because the original recruitment frames differed in zone coverage.

For variables containing left-censored observations and sufficient detectable information, between-group differences were evaluated using censoring-aware rank-based procedures [41,42]. Uncensored two-group comparisons were performed using Wilcoxon rank-sum tests, whereas comparisons involving more than two groups used Kruskal–Wallis tests followed, where appropriate, by pairwise Wilcoxon rank-sum comparisons. Residential-zone comparisons followed the corresponding censored or uncensored framework according to the analytical characteristics of each element.

Eligibility for primary inferential testing was determined from the degree of censoring and the amount of detectable information available in the comparison dataset. Descriptive estimability within an individual subgroup was considered separately from eligibility of the complete dataset for a censoring-aware comparison; detailed decision criteria and implementation procedures are provided in Supplementary Methods S3.

Raw *p*-values were adjusted within the relevant families of element-wise comparisons using the Benjamini–Hochberg false-discovery-rate procedure [32]. Adjusted *q*-values < 0.05 were regarded as FDR-robust, whereas nominal associations that did not remain significant after adjustment were treated as exploratory. Interpretation considered censoring, detection frequency, effect direction, and distributional consistency in addition to statistical significance.

#### 2.6.3. Hair–Hair Correlation Analyses

Hair–hair associations were examined using Spearman rank correlations. To minimise bias associated with nondetect handling, primary correlation matrices were restricted to elements without censored observations in the relevant analytical population. The full-cohort primary matrix therefore included Fe, Mo, and Se, whereas the adolescent matrix additionally included Mg. Benjamini–Hochberg adjustment was applied to pairwise correlations within each matrix [32].

An exploratory adolescent matrix additionally included Ca, which showed ≤10% censoring; LoD-bounded Ca observations were retained with their induced rank ties. This analysis is presented in Supplementary Figure S3 and was not treated as primary censored-data inference. Correlation coefficients were interpreted only as co-occurrence signals and not as evidence of physiological coupling, a common exposure source, causality, or environmental source apportionment.

#### 2.6.4. Zone-Level Hair–Soil Contextualisation

Published historical topsoil data from Alcalá de Henares were used as secondary ecological context for Co, Fe, Mo, and Ni, the elements represented in both datasets [22]. Soil observations were reduced to independent sampling-location or composite units before zonal summarisation, yielding 97 independent locations for Co, Fe, and Mo and 74 for Ni. Hair and soil distributions were then summarised separately within the corresponding residential zones using medians and interquartile ranges where estimable, together with detection and censoring information where required. Where sufficient zonal medians were available, Spearman’s rho was calculated descriptively across the three child zones or four adolescent zones. No inferential *p*-values were attached to these ecological coefficients, and no soil value was assigned to individual participants. The analysis was therefore interpreted only as zone-level environmental context and not as evidence of individual exposure, source attribution, or direct soil-to-hair transfer. Further details of the soil-data reduction and contextualisation workflow are provided in Supplementary Methods S5.

#### 2.6.5. External Contextualisation and Interpretive Scope

External comparisons were used only for screening-level contextualisation, with priority given to Spanish paediatric studies and age-relevant population distributions. Cross-study differences were interpreted cautiously because of variation in population characteristics, hair preparation, analytical methods, analytical sensitivity, and treatment of nondetects. Because harmonised health-based guidance values for scalp hair are generally unavailable for the elements considered, external values were not used to diagnose nutritional deficiency or excess, estimate internal dose, or infer adverse health effects. FFQ and topsoil information were likewise treated only as complementary population or ecological context and not as participant-level determinants of hair concentrations.

#### 2.6.6. Software and Reproducibility

All statistical analyses were conducted in R. Detailed software-package versions, censoring-aware implementation checks, analytical decision criteria, and reproducibility information are provided in Supplementary Methods S3 and, for the child Ni harmonisation audit, Supplementary Methods S4 [36,43].

## 3. Results

Analytical detectability, developmental-stage differences, sex-related patterns, and residential-zone contrasts are summarised below. Concentrations are expressed as µg g^−1^ and are reported to a maximum of three significant figures. Unless otherwise stated, Ni results refer to the final harmonised censoring-aware analytical field. Detailed age- and sex-stratified analytical distributions are provided in Supplementary Tables S5–S10. B and Li were too highly censored to support stable distributional or subgroup inference and are therefore described primarily in terms of detectability.

### 3.1. Analytical Detectability and Overall Element Profiles

Analytical detectability differed substantially across the nine-element panel and between developmental stages (Table 1). Fe, Mo, and Se were quantified in all 120 children and all 97 adolescents. Mg was also consistently detected, with concentrations above the LoD in 111/120 children (92.5%) and all adolescents. Ca detectability increased markedly from 64/120 children (53.3%) to 95/97 adolescents (97.9%), whereas Co showed similar detection frequencies in children (74.2%) and adolescents (76.3%). Ni was detected in 58/120 children (48.3%) and 55/97 adolescents (56.7%).

**Figure 1:**
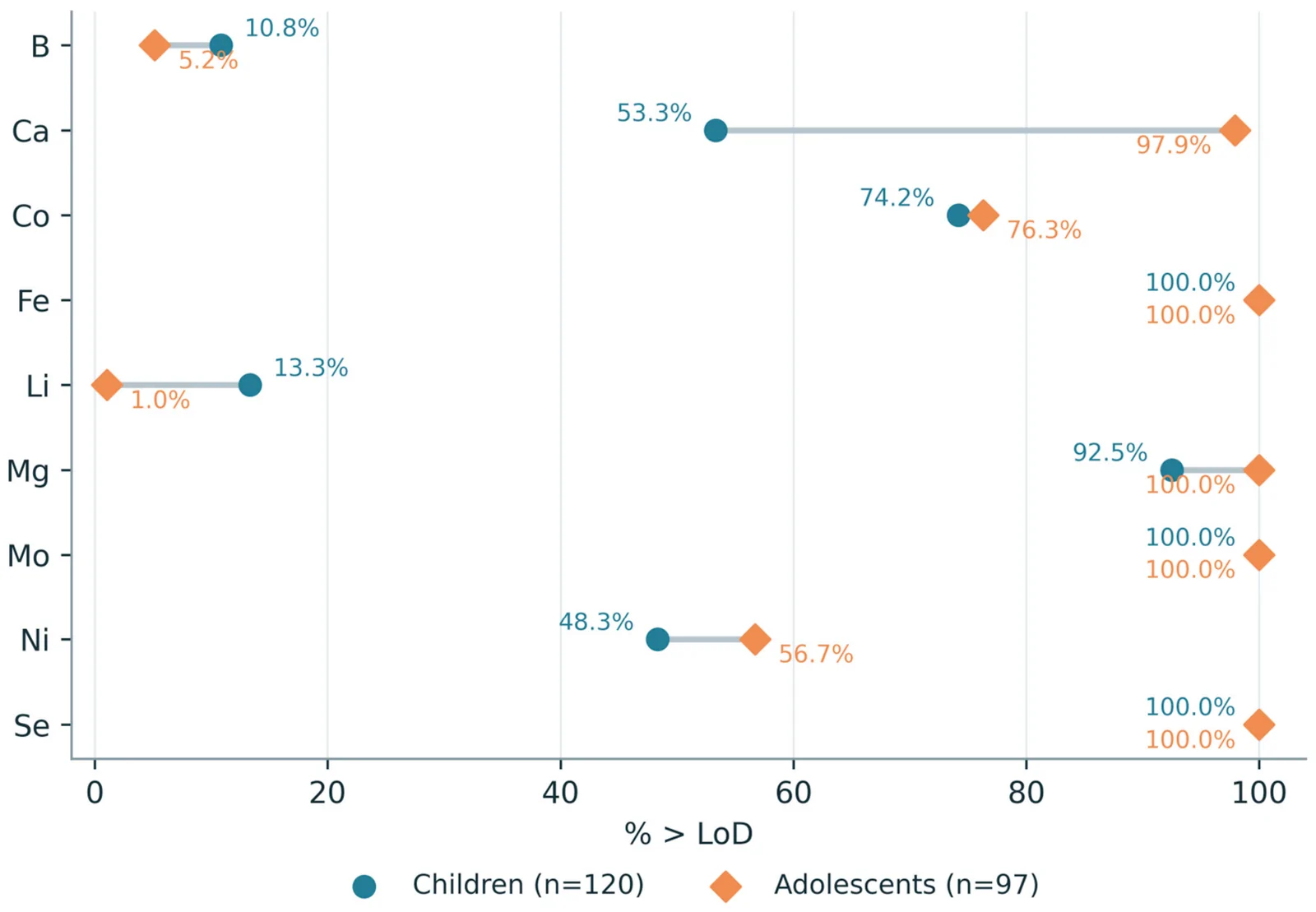
Analytical detectability of the nine-element scalp-hair panel in children and adolescents. The paired dot plot shows the percentage of samples with concentrations above the age-stratum-specific analytical limit of detection (LoD) in children aged 6–9 years (*n* = 120) and adolescents aged 13–16 years (*n* = 97). B and Li were highly censored, whereas Fe, Mo, and Se were quantified in all participants, and Mg was quantified in 92.5% of children and 100% of adolescents. Percentages represent analytical detectability only and should not be interpreted as concentration differences.

In contrast, B and Li provided limited quantitative information. B was detected in 13/120 children (10.8%) and 5/97 adolescents (5.15%), while Li was detected in 16/120 children (13.3%) and only 1/97 adolescents (1.03%). Stable central or upper-tail estimates were therefore not derived for these two elements. These element-specific analytical-detectability profiles are illustrated in Figure 1.

Among the elements supporting distributional estimation, Ca and Mg showed prominent developmental shifts. Median Ca increased from 168 µg g^−1^ in children to 325 µg g^−1^ in adolescents, and median Mg increased from 9.05 to 24.0 µg g^−1^. Ni similarly showed a higher censoring-aware median in adolescents (0.169 µg g^−1^) than in children (0.0597 µg g^−1^). In contrast, median Fe, Co, and Mo concentrations were lower in adolescents, whereas Se remained similar between developmental stages. Several elements also showed broad upper tails, particularly Ca, Mg, Ni, and Fe.

### 3.2. Sex-Related Differences

Sex-related differences were observed within both developmental strata, although the affected elements and magnitude of the contrasts differed (Table 2).

Among children, girls showed higher Ca, Co, Fe, Mg, and Ni concentration distributions than boys, and all five contrasts remained significant after Benjamini–Hochberg adjustment. The strongest sex-related contrast involved Ca (*q* = 3.92 × 10^−9^). Ca was detected in only 10/50 boys, precluding estimation of a stable boys’ median, compared with 54/70 detections and a median of 222 µg g^−1^ in girls. All censored and uncensored observations from both groups nevertheless contributed to the censoring-aware comparison.

Mg was also substantially higher in girls, with medians of 13.4 versus 5.94 µg g^−1^ in boys (*q* = 6.37 × 10^−7^). Ni showed substantially greater detectability in girls (46/70) than boys (12/50), together with a higher censoring-aware estimated median (0.117 vs. 0.00568 µg g^−1^; *q* = 7.98 × 10^−5^). The latter value is a model-derived distributional estimate and should not be interpreted as a directly observed concentration. Smaller but FDR-robust differences were observed for Co (0.00755 vs. 0.00474 µg g^−1^; *q* = 8.90 × 10^−4^) and Fe (7.86 vs. 6.59 µg g^−1^; *q* = 0.0273). Mo and Se did not differ significantly by sex.

Among adolescents, females had higher median Ca, Co, and Fe concentrations than males. Median Ca was 375 µg g^−1^ in females compared with 168 µg g^−1^ in males (*q* = 0.0353), median Co was 0.00528 versus 0.00220 µg g^−1^ (*q* = 0.00120), and median Fe was 5.60 versus 4.52 µg g^−1^ (*q* = 0.00120). Ni also differed significantly by sex, with substantially greater detectability in females: concentrations exceeded the LoD in 47/68 females compared with only 8/29 males. Although the number of male detections did not support estimation of a stable male median, the complete censoring-aware distributions differed significantly (*q* = 0.0353). Mg, Mo, and Se showed no FDR-robust sex-related differences among adolescents. B and Li were not subjected to primary sex-related inference because of their high censoring.

### 3.3. Differences Between Children and Adolescents

Developmental-stage comparisons revealed divergent rather than uniform age-related patterns across the element panel (Table 3). Adolescents had higher median concentrations of Ca, Mg, and Ni, whereas median Fe, Co, and Mo concentrations were lower than in children.

The strongest age-related contrast involved Mg, whose median increased from 9.05 µg g^−1^ in children to 24.0 µg g^−1^ in adolescents (*q* = 8.91 × 10^−19^). Ca increased from a median of 168 to 325 µg g^−1^ (*q* = 1.43 × 10^−9^), accompanied by an increase in detectability from 53.3% to 97.9%. Median Ni increased from 0.0597 to 0.169 µg g^−1^ (*q* = 2.10 × 10^−5^).

In the opposite direction, median Fe decreased from 7.54 to 5.16 µg g^−1^ (*q* = 2.28 × 10^−11^), median Co from 0.00632 to 0.00381 µg g^−1^ (*q* = 0.00978), and median Mo from 0.0298 to 0.0231 µg g^−1^ (*q* = 9.31 × 10^−6^). Se was comparatively stable, with medians of 0.318 and 0.323 µg g^−1^ in children and adolescents, respectively (*q* = 0.563). B and Li were not subjected to primary developmental-stage inference because of insufficient detectable information.

### 3.4. Residential-Zone Differences

Residential-zone patterns differed markedly between developmental strata. Detailed child and adolescent residential-zone results are provided in Supplementary Tables S11 and S12, respectively. No overall child-zone comparison remained significant after FDR adjustment. Se showed the strongest unadjusted child-zone pattern (*p* = 0.00800), but this did not remain FDR-robust (*q* = 0.0640).

**Figure 2:**
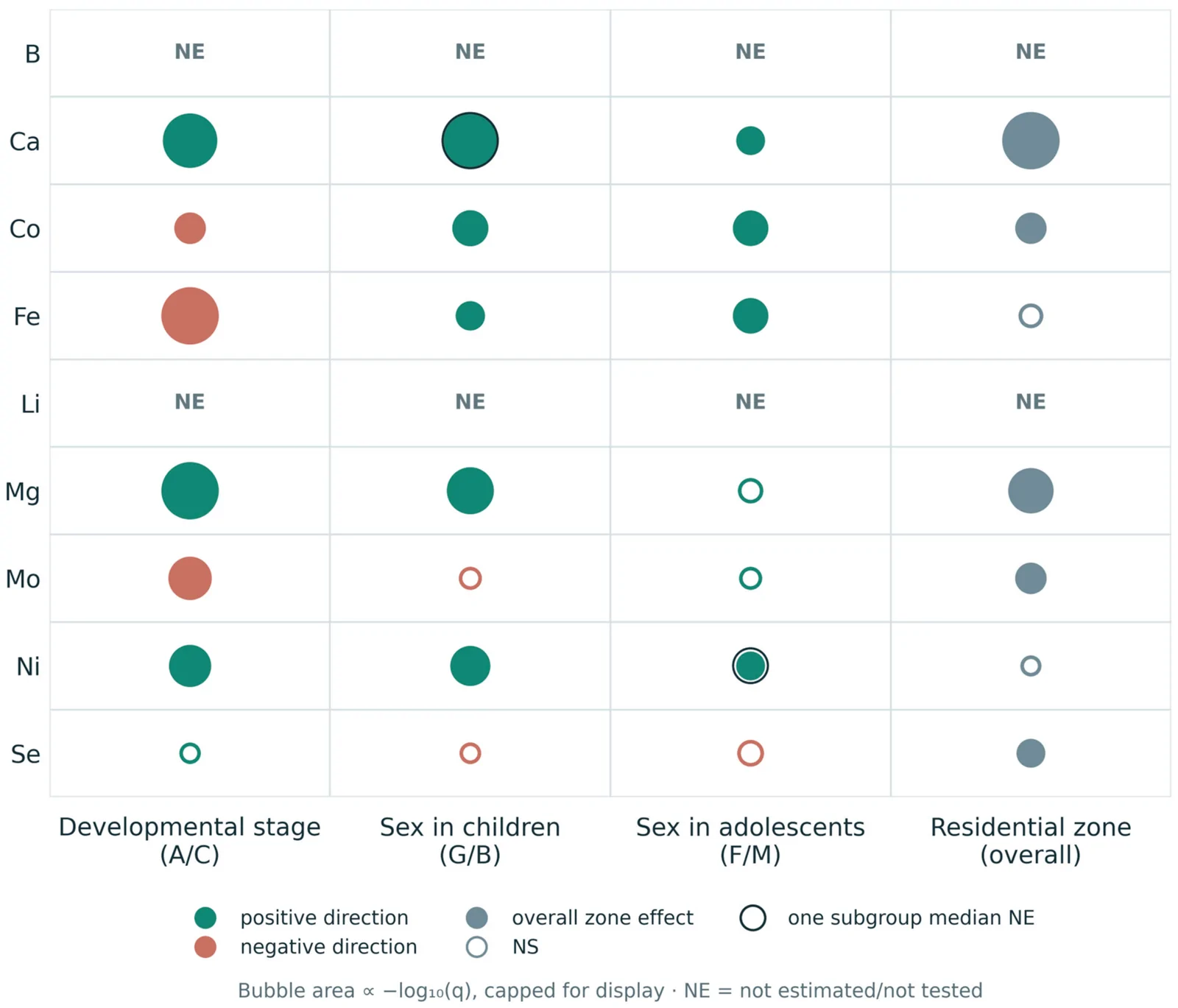
Integrated summary of developmental-stage, sex-related, and adolescent residential-zone comparisons across the nine-element panel. Each column represents one comparison. For binary contrasts, green indicates higher values in adolescents versus children (A/C), girls versus boys (G/B), or females versus males (F/M), whereas rust indicates lower values. Larger circles indicate smaller FDR-adjusted *q*-values. Grey circles indicate a significant overall residential-zone effect without assigning a single direction. Open circles indicate non-significant comparisons, ringed circles identify significant comparisons for which one subgroup median was not estimable, and NE denotes comparisons not evaluated because of insufficient analytical information/high censoring. Bubble area is proportional to −log_10_(*q*) and capped for display.

In adolescents, Ca, Co, Mg, Mo, and Se differed significantly among the four residential/environmental zones after FDR adjustment (Table 4). An integrated overview of the developmental-stage, sex-related, and adolescent residential-zone evidence across the elemental panel is provided in Figure 2. Figure 2 is intended as a compact evidence summary rather than as a concentration plot. Each column represents a different comparison; larger circles indicate stronger FDR-adjusted statistical evidence (smaller *q*-values), colour indicates direction for binary contrasts, grey denotes a significant overall residential-zone effect without assigning a single direction, and open or NE symbols identify non-significant or non-evaluated comparisons, respectively. The strongest spatial signals involved Ca and Mg and were characterised by a marked upward displacement in Zone IV.

**Figure 3:**
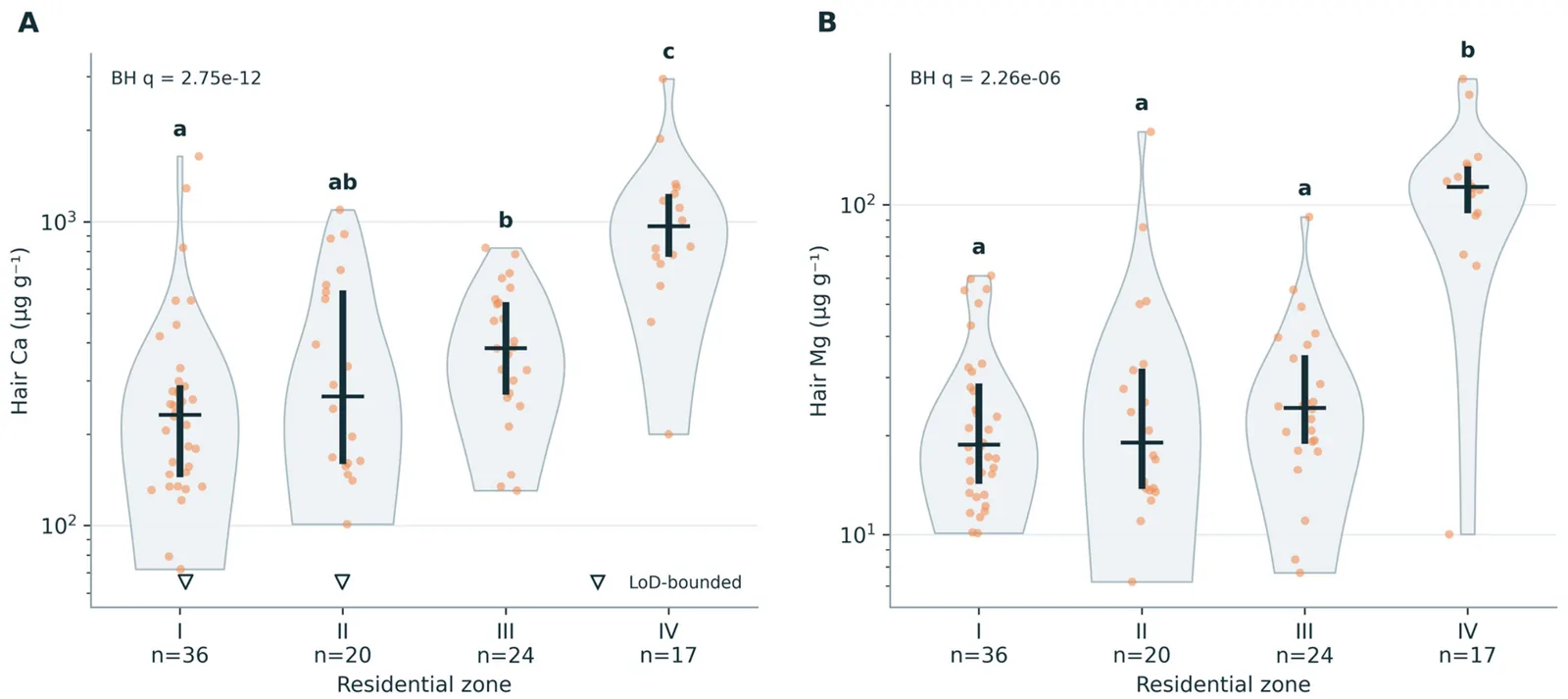
Residential-zone distributions of scalp-hair calcium (Ca; panel (**A**)) and magnesium (Mg; panel (**B**)) in adolescents. Violin plots show individual observations across Zones I–IV (*n* = 36, 20, 24, and 17, respectively), with black summary bars indicating the median and interquartile range; concentration axes are logarithmic. For Ca, the two left-censored observations are plotted at their analytical LoDs only as open downward triangles and are interpreted as LoD-bounded values rather than observed concentrations. Overall q values are Benjamini–Hochberg-adjusted. Within each element, zones sharing a lowercase letter did not differ after pairwise Benjamini–Hochberg adjustment: for Ca, Zone I = a, Zone II = ab, Zone III = b, and Zone IV = c; for Mg, Zones I–III = a and Zone IV = b.

Median Ca increased from 232 µg g^−1^ in Zone I and 267 µg g^−1^ in Zone II to 385 µg g^−1^ in Zone III and 967 µg g^−1^ in Zone IV (*q* = 2.75 × 10^−12^). Adjusted pairwise comparisons separated Zone IV from Zones I–III, while Zone III also exceeded Zone I. Median Mg was 18.8, 19.1, and 24.3 µg g^−1^ in Zones I–III, respectively, but increased to 113 µg g^−1^ in Zone IV (*q* = 2.26 × 10^−6^). Zone IV differed from each of the other three zones, whereas Zones I–III were not distinguishable from one another (Figure 3).

Co also showed significant spatial heterogeneity (*q* = 0.0111). Zone I had the lowest median concentration (0.00171 µg g^−1^) and differed from Zones II–IV, which did not differ significantly from one another. Mo showed a different pattern (*q* = 0.0106), with Zone III lower than Zones I and II and Zone IV occupying an intermediate post hoc position. Se also differed among zones (*q* = 0.0361), with Zone IV higher than Zone I.

No significant overall residential-zone effect was observed for Fe (*q* = 0.185) or Ni (*q* = 0.627). B and Li remained too highly censored for primary spatial inference. Thus, the developmental- and sex-related Ni differences occurred without evidence of a corresponding residential-zone gradient in this dataset.

### 3.5. Element Co-Occurrence and Ecological Hair–Soil Context

In the primary full-cohort analysis, Fe and Mo showed a moderate positive association (Spearman’s *ρ* = 0.368; BH-adjusted *q* = 7.24 × 10^−8^), whereas Fe–Se and Mo–Se associations were not FDR-significant (Supplementary Figure S4). Within adolescents, Fe was positively associated with Mg (*ρ* = 0.377; *q* = 8.50 × 10^−4^), while no other pair among Fe, Mg, Mo, and Se remained significant after multiple-testing adjustment.

The exploratory adolescent analysis that additionally included Ca identified a strong Ca–Mg association (*ρ* = 0.883; *q* = 6.86 × 10^−32^) and a positive Ca–Fe association (*ρ* = 0.501; *q* = 8.80 × 10^−7^). Fe–Mg also remained significant within this expanded matrix (*ρ* = 0.377; q = 4.72 × 10^−4^). Because the two LoD-bounded Ca observations were retained with their induced rank ties, these results were treated as exploratory rather than primary censored-data inference (Supplementary Figure S3).

Zone-level comparison with historical topsoil was restricted to Fe, Co, Mo, and Ni. Fe and Co showed identical relative zonal ordering across the three child zones (descriptive *ρ* = 1.00) and strong rank concordance across the four adolescent zones (*ρ* = 0.800). No inferential *p*-values were assigned because these coefficients were based on only three or four ecological units and the hair and soil datasets were not individually paired. Mo correspondence could not be estimated because soil censoring ranged from 87.5% to 100% across zones, while Ni medians were simultaneously estimable in too few hair–soil zone combinations to support a meaningful rank coefficient (Supplementary Figure S5). These findings therefore represent ecological zonal concordance for Fe and Co rather than evidence of participant-level exposure or direct soil-to-hair transfer.

## 4. Discussion

Scalp hair provides a practical matrix for retrospective paediatric biomonitoring because collection is non-invasive, sample requirements are modest, and archived specimens can support reconstruction of historical population profiles. Its interpretation is nevertheless element-specific because measured concentrations may integrate endogenous incorporation during keratinisation with exogenous contributions from water, household dust, airborne particles, and other surface-related sources. Within the standardised washing, digestion, ICP–MS, and QA/QC framework applied here, the present dataset is therefore most appropriately interpreted as a historical population-level screening profile rather than as a direct measure of nutritional adequacy, internal dose, or health status [25,26,44].

The principal findings were strongly structured by developmental stage, sex, and residential setting. Fe, Mg, Mo, and Se provided the most complete analytical distributions, whereas B and Li were dominated by nondetects. Adolescents had higher median Ca, Mg, and Ni concentrations but lower Fe, Co, and Mo concentrations than children, while Se remained comparatively stable. Sex-related differences were evident in both developmental strata, and residential heterogeneity was substantially stronger among adolescents, particularly for Ca and Mg in the mixed residential–industrial Zone IV. Together, these findings support an element-specific interpretation along a homeostasis-to-exposure continuum rather than a single nutritional, physiological, or environmental model.

### 4.1. Principal Findings and Comparison with Spanish Paediatric Studies

The present study extends the limited Spanish literature on multi-element paediatric hair profiles by evaluating children and adolescents from the same municipality within a common analytical campaign and unified censoring-aware framework. Llorente Ballesteros et al. [18] reported reference distributions for several trace elements in hair from children and adolescents in the Madrid region, including Co, Fe, Mo, Ni, and Se, whereas Ruiz et al. [19] subsequently quantified 28 elements in children from eastern Spain and proposed provisional reference values for several analytes overlapping with the present panel. These studies provide the most relevant national comparators, although differences in population structure, sampling, hair preparation, analytical sensitivity, and nondetect handling preclude direct equivalence.

Relative to the children studied by Ruiz et al. [19], children from Alcalá showed lower median concentrations of Ca, Mg, Ni, and Se. Median Ca was 168 µg g^−1^ in Alcalá compared with approximately 279 µg g^−1^ in the Mediterranean study, while Mg was 9.05 versus approximately 18.7 µg g^−1^. Corresponding Ni medians were approximately 0.060 and 0.216 µg g^−1^, and Se medians approximately 0.318 and 0.480 µg g^−1^. Fe was slightly lower but remained within the same order of magnitude, whereas Co was broadly comparable. The Alcalá profile should therefore not be characterised as uniformly elevated or depleted relative to other Spanish populations.

Comparison with the Madrid study [18] leads to a similar interpretation. Median Fe concentrations in Alcalá were lower in both developmental strata than the approximately 15.5 µg g^−1^ reported in Madrid. Ni and Se were also lower, and adolescent Mo was below the Madrid comparator. After unit conversion, median Co concentrations in Alcalá were approximately 6.32 ng g^−1^ in children and 3.81 ng g^−1^ in adolescents, compared with approximately 14.5 ng g^−1^ in the Madrid study. Against this generally moderate trace-element profile, the most distinctive feature of the present dataset was the selective developmental and spatial behaviour of Ca and Mg.

Upper-tail descriptors reinforce this pattern. The Ca P95 increased from 509 µg g^−1^ in children to 1.29 × 10^3^ µg g^−1^ in adolescents, while the Mg P95 increased from 36.6 to 131 µg g^−1^ (Table 1). These upper-tail values describe the historical population distribution and should not be interpreted as clinical or nutritional thresholds.

The harmonisation of child Ni provides an additional methodological insight without altering the primary biological focus. The historical substitution-based field yielded a median of 0.230 µg g^−1^ and P95 of 1.04 µg g^−1^, whereas the final censoring-aware field yielded a median of 0.0597 µg g^−1^ and P95 of 0.586 µg g^−1^; the latter also identified a robust sex-related difference (*q* = 7.98 × 10^−5^) that was not apparent in the legacy analysis (Supplementary Table S4). Because both fields derive from the same underlying child cohort, this comparison illustrates how nondetect handling can influence both distributional descriptors and inferential conclusions when censoring is substantial [34,35,36,40].

### 4.2. Developmental-Stage and Sex-Related Patterns

Developmental-stage comparisons revealed divergent rather than uniform patterns across the element panel (Table 3). Median Ca increased from 168 to 325 µg g^−1^, Mg from 9.05 to 24.0 µg g^−1^, and Ni from 0.0597 to 0.169 µg g^−1^ between children and adolescents. In contrast, Fe decreased from 7.54 to 5.16 µg g^−1^, Co from 0.00632 to 0.00381 µg g^−1^, and Mo from 0.0298 to 0.0231 µg g^−1^, while Se remained almost unchanged at 0.318 and 0.323 µg g^−1^, respectively. This divergent behaviour argues strongly against interpreting the complete panel through a single developmental mechanism.

Ca and Mg showed the clearest developmental increases. Such a pattern is biologically compatible with the accelerated skeletal growth, bone-mineral accretion, changing body composition, and increased mineral requirements associated with puberty [6,7]. However, biological plausibility does not establish that hair Ca or Mg directly represent skeletal stores or systemic mineral status. Skröder et al. [44] reported limited correspondence between hair Ca and Mg and conventional internal biomarkers in children and observed changes along the proximal-to-distal hair axis, compatible with segment-related accumulation or external contribution. Conversely, Marcinek et al. [45] identified an association between dietary Ca intake and hair Ca in young children, while Vanaelst et al. [46] reported associations between hair Ca and Mg and selected anthropometric or metabolic variables in schoolgirls. Experimental evidence also indicates that ambient particulate exposure can alter hair-element concentrations even after washing [47]. The most defensible interpretation is therefore that hair Ca and Mg integrate developmental physiology, dietary background, hair-matrix behaviour, and potential environmental contribution rather than serving as direct measures of mineral status. The experimental evidence for persistent ambient-particle interference supports particular caution when interpreting spatial hair-element differences.

Fe showed the opposite developmental pattern, with median concentrations decreasing from 7.54 µg g^−1^ in children to 5.16 µg g^−1^ in adolescents (*q* = 2.28 × 10^−11^). Adolescence is a period of substantially increased iron requirements associated with rapid growth, expansion of blood volume and lean tissue, and, in females, menstrual iron losses [8]. This biological context is particularly relevant in Spain: a population-based study of 405 healthy Spanish adolescents aged 12–16 years reported iron deficiency in 13.3% of participants, although iron-deficiency anaemia was much less frequent [48]. These observations support adolescence as a physiologically important period for iron metabolism but do not establish that the lower hair Fe observed here reflects iron deficiency. Hair Fe is not a validated surrogate for ferritin, haemoglobin, or systemic iron stores [44], and endogenous incorporation and particulate deposition cannot be separated completely in the present design.

Sex-related findings also argue against a simple nutritional interpretation (Table 2). Among children, girls showed higher Ca, Co, Fe, Mg, and Ni distributions than boys; Ca showed the strongest contrast, with only 10/50 boys above the LoD compared with 54/70 girls, while median Mg was 13.4 µg g^−1^ in girls versus 5.94 µg g^−1^ in boys. Ni was detected in 46/70 girls but only 12/50 boys. Among adolescents, females had higher Ca, Co, and Fe, and Ni again showed a significant sex-related distributional difference, with 47/68 females but only 8/29 males above the LoD. Similar sex-associated variation has been reported in Spanish and other populations [18,19,49,50].

These differences may reflect interacting determinants including developmental physiology, pubertal stage, hair growth and structure, sebaceous activity, hair length, hygiene practices, consumer-product use, jewellery or piercing, and contact with metal-containing objects. Restriction to participants with naturally dark, untreated hair reduced variability attributable to dyeing and chemical treatments but could not eliminate these biological and behavioural influences. Sex should therefore be treated as an important stratification variable in paediatric hair biomonitoring rather than as a mechanistic explanation by itself.

Co and Mo were also lower in adolescents. Co is nutritionally relevant through cobalamin but may additionally reflect geological or anthropogenic sources [11], whereas Mo is essential through the molybdenum cofactor but lacks an established interpretation as a scalp-hair marker of nutritional status [10]. Their opposite direction relative to Ca and Mg further demonstrates that developmental changes in hair-element profiles are element-specific.

Se differed from the other trace elements because its median remained almost unchanged between developmental stages. This relative stability is compatible with tighter physiological regulation and with evidence that hair Se may show closer correspondence than many other elements with conventional biological matrices [44]. Nevertheless, hair and blood or serum remain non-interchangeable matrices [51,52], and neither developmental stability nor the modest adolescent zone effect can be translated directly into selenium nutritional status.

Ni lies closer to the exposure-related end of the interpretive continuum. It was higher in adolescents than children and showed significant sex-related differences in both strata, but importantly did not show a significant residential-zone effect (*q* = 0.627). Tamburo et al. [50] similarly reported higher hair Ni concentrations in adolescent girls than boys across multiple study sites. Potential determinants include diet, external deposition, jewellery or piercing, metal contact, consumer products, and hair characteristics. Female sex and ear piercing have also been associated with nickel sensitisation [53], although sensitisation was not assessed here, and hair Ni cannot be used to infer allergic risk. The present data therefore support mixed behavioural, contact-related, and exposure-related determinants rather than a demonstrable residential Ni source.

B and Li provided considerably less quantitative information. B was censored in 89.2% of children and 94.8% of adolescents, while Li was censored in 86.7% and 99.0%, respectively. Their potential determinants include diet, drinking water, environmental media, and consumer products [13,14,15], with B additionally associated with some arts-and-crafts materials used by children [13,14]. Under the present analytical conditions, however, these elements are informative principally through detectability rather than stable population distributions. Their behaviour contrasts with that of consistently quantified Fe, Mg, Mo, and Se and further demonstrates the element-specific suitability of hair for biomonitoring [54].

The aggregated FFQ provides only broad dietary context and cannot resolve these developmental, sex, or spatial findings. Although commonly consumed foods were compatible with a broadly typical local diet, the questionnaire lacked participant-level intake, portion size, supplement use, detailed food composition, and drinking-water information. Importantly, its broad age–sex patterns did not parallel the pronounced Ca and Mg elevation in adolescent Zone IV. Detailed FFQ results are therefore retained in the Supplementary Materials and are not interpreted as evidence of specific diet–hair relationships [55,56,57].

### 4.3. Residential Heterogeneity, Element Co-Occurrence, and Environmental Context

Residential heterogeneity was substantially stronger among adolescents than children. No child-zone comparison remained significant after FDR adjustment, whereas Ca, Co, Mg, Mo, and Se differed among adolescent zones (Table 4). The spatial signal was therefore selective both by developmental stratum and by element rather than indicative of generalised multi-element enrichment throughout the municipality.

The strongest residential pattern involved Ca and Mg. Median adolescent Ca was 232, 267, 385, and 967 µg g^−1^ in Zones I–IV, respectively (*q* = 2.75 × 10^−12^), while corresponding Mg medians were 18.8, 19.1, 24.3, and 113 µg g^−1^ (*q* = 2.26 × 10^−6^). Thus, the Zone IV median was approximately 2.5–4-fold higher for Ca and 4.7–6-fold higher for Mg than in the other zones. The broad Zone IV distributions shown in Figure 3 indicate an upward displacement affecting the group rather than a signal produced solely by a few extreme observations.

Because these contrasts occurred within the adolescent stratum, they cannot be explained solely by the overall child–adolescent developmental difference, although residual differences in age and pubertal stage within the 13–16-year range remain possible. No local environmental measurements of Ca or Mg were available, and the observed Zone IV pattern should therefore be regarded as hypothesis-generating rather than as evidence of environmental exposure. The present design cannot distinguish environmental, physiological, dietary, behavioural, or other explanations for this spatial pattern and does not support attribution to a specific industrial or environmental source. Targeted studies incorporating participant-linked environmental measurements would be required to investigate its origin.

The remaining zonal findings followed different configurations. Co was lowest in Zone I and higher in Zones II–IV, suggesting differentiation between the lower-density greener residential setting and the more urbanised zones rather than a monotonic industrial gradient. Mo was lowest in Zone III, a pattern inconsistent with simple traffic-related enrichment. Se was modestly higher in Zone IV than Zone I. In contrast, Fe and Ni showed no FDR-robust residential-zone differences. The element-specific nature of these patterns argues against a uniform environmental elevation in Zone IV.

Hair–hair and hair–soil associations were retained only as secondary, non-mechanistic context. Positive associations among Fe, Mo, Mg, and, exploratorily, Ca were observed, with the strong Ca–Mg co-occurrence broadly paralleling their residential-zone pattern (Supplementary Figures S3 and S4). However, these correlations cannot distinguish physiological co-variation from shared spatial structure or other common determinants. Likewise, the zone-level hair–soil concordance observed for Fe and Co was based on only three or four ecological units and therefore indicates only broad compatibility between the historical datasets rather than participant-level exposure or direct soil-to-hair transfer; Mo and Ni could not be evaluated robustly because of limited estimability (Supplementary Figure S5). These secondary analyses consequently do not alter the primary population-level interpretation.

### 4.4. Historical Archival Storage and Interpretation

The 24-year interval between collection in 2001 and instrumental analysis in 2025 is an important feature of this study. Hair is a highly keratinised matrix that can remain physically preserved for prolonged periods and has long been used for retrospective elemental investigations [26]. Direct evidence of analytical feasibility comes from Thomas et al. [58], who quantified 33 inorganic elements by ICP–MS in 138 human hair strands collected in the French Alps around 1880 and subsequently preserved in a museum collection.

More controlled evidence is provided by recent work on a human-hair certified reference material. Yamakawa et al. [59] reported long-term stability of a human-hair CRM for more than 10 years, with certified or reference information encompassing several trace elements and major elements including Ca, Mg, Fe, and Se. This provides experimental support for the inherent suitability of keratinised hair as a long-term elemental reference matrix under controlled conditions.

Neither observation, however, demonstrates that the absolute concentration of every analyte remains quantitatively unchanged over 24 years under the specific storage conditions of the present cohort. Element-specific redistribution, external contamination, humidity, handling, and interaction with storage materials cannot be excluded retrospectively. This distinction is particularly relevant for elements susceptible to exogenous deposition and for analytes measured close to their LoDs [26,47].

In the present study, samples had been retained dry in paper envelopes, and all archived specimens were processed together using a common modern washing, digestion, ICP–MS, internal-standard, CRM, and QA/QC workflow [23,24]. These features strengthen internal comparability among age, sex, and residential groups. However, no replicate aliquots analysed in 2001, intermediate analytical time points, or controlled long-term storage experiment were available. The data can therefore support a historical population profile and comparisons within the archived cohort, whereas absolute comparison with freshly collected contemporary hair should acknowledge possible unquantified storage effects.

### 4.5. Strengths and Limitations

The study has several strengths. It evaluates a rare archived paediatric cohort of 217 participants from a single urban–industrial municipality and includes two clearly defined developmental strata. All specimens were analysed in the same contemporary analytical campaign using a common decontamination, digestion, ICP–MS, internal-standard, CRM, and QA/QC workflow, strengthening internal comparisons by age, sex, and residential zone. Element-specific analytical recoveries were also characterised rather than relying solely on nominal CRM concentrations.

A further strength is the explicit treatment of left-censored observations. Nondetects were not replaced by arbitrary fixed values in the principal analytical field; child Ni was harmonised from the analytical source; statistical procedures were selected according to censoring intensity; and FDR correction was applied to the relevant comparison families. Primary and exploratory correlations were distinguished according to analytical completeness, and the environmental analysis used independent soil locations and ecological zone summaries rather than repeated assignment of environmental values to participants [34,35,36,40].

Several limitations nevertheless delimit interpretation. Scalp hair is not a universally standardised marker of internal dose or nutritional status, and endogenous incorporation cannot be separated completely from residual exogenous deposition. No paired blood or urine measurements, washing-effluent analyses, repeated hair samples, or sequential segment analyses were available. The historical storage interval introduces additional uncertainty, as discussed above, although common storage and analytical processing strengthen within-cohort comparability.

The study was cross-sectional and school-based, and children and adolescents had different residential-zone coverage; specifically, Zone III was absent from the child sample. Pubertal stage, socioeconomic characteristics, detailed hair length and hygiene information, jewellery or piercing, household occupations, individual mobility, and exact residential proximity to potential sources were not available for multivariable adjustment. Consequently, age-, sex-, and zone-associated differences may include residual biological, behavioural, or compositional influences.

The topsoil and hair datasets were not individually paired, and their comparison involved only three or four ecological zones. It cannot distinguish ingestion, inhalation, dermal contact, and external deposition or quantify participant-specific uptake. Local topsoil information was unavailable for Ca and Mg, the elements showing the strongest adolescent residential pattern. The FFQ likewise consisted of aggregated age–sex frequency data and could not be linked to individual hair concentrations or used to estimate element intake.

Finally, analytical sensitivity differed substantially among elements and between some strata. B and Li did not support stable central estimates or subgroup inference, while Co and Ni remained appreciably censored in several comparisons. Differences in LoDs between children and adolescents should therefore be considered when interpreting detectability, and model-derived summaries of censored distributions should not be treated as directly observed concentrations. Population percentiles reported here are historical screening descriptors rather than clinical, nutritional, or toxicological thresholds.

Within these boundaries, the principal contribution of the study is the demonstration that archived paediatric scalp hair can provide a coherent historical population profile when developmental physiology, sex, hair-matrix behaviour, environmental plausibility, analytical detectability, and censoring-aware inference are considered jointly. The study identifies reproducible developmental and sex-related patterns, a particularly strong adolescent Ca–Mg residential signal, and clear limits to what can be inferred from dietary or ecological soil information without participant-level linkage.

## 5. Conclusions

Scalp hair provided a useful retrospective screening matrix for characterising nutritionally relevant and mixed-source elements in children and adolescents from urban–industrial Alcalá de Henares. Its analytical and interpretive value was strongly element-specific. Fe, Mg, Mo, and Se supported consistent distributional characterisation, whereas Ca, Co, and Ni required explicit consideration of censoring, and B and Li were informative principally through detectability. The numerical audit of child Ni further demonstrated that substitution-based handling of nondetects can materially alter central and upper-tail estimates and may also affect between-group inference. Population percentiles derived from the better-characterised elements should therefore be regarded as historical screening comparators rather than clinical, nutritional, or toxicological thresholds.

Age- and sex-related patterns were not uniform across the element panel. Girls showed higher distributions of several elements in both age strata, while adolescents had higher Ca, Mg, and Ni but lower Fe, Co, and Mo than children. The most pronounced spatial signal was the marked elevation of Ca and Mg among adolescents residing in the mixed residential–industrial zone. This pattern was not paralleled by the aggregated food-frequency results; however, in the absence of corresponding environmental Ca and Mg measurements, it should be regarded as a hypothesis-generating zone-associated signal rather than as evidence of environmental exposure. Its origin cannot be resolved from the present data and requires targeted participant-linked environmental investigation. Hair–hair associations should similarly be interpreted as co-occurrence patterns, while the observed zonal correspondence between hair and topsoil Fe and Co remains ecological and does not demonstrate participant-level exposure or soil-to-hair transfer.

Overall, paediatric scalp hair should not be interpreted as a direct proxy for nutritional adequacy, systemic dose, deficiency, toxicity, or health risk. Its principal value lies in contextual population biomonitoring, particularly when analytical censoring, age, sex, hair-matrix behaviour, and environmental heterogeneity are considered jointly. The Alcalá cohort provides a valuable historical Spanish baseline and supports future studies combining contemporary hair sampling with paired conventional biomarkers, individual dietary and behavioural information, drinking-water and dust measurements, and participant-linked environmental data.

## Figures and Tables

**Table 1:** Analytical detectability and overall scalp-hair element distributions by developmental stage (concentrations in µg g^−1^).

Element	Children *n* > LoD (%)	Children Median (IQR), µg g^−1^	Children P95, µg g^−1^	Adolescents *n* > LoD (%)	Adolescents Median (IQR), µg g^−1^	Adolescents P95, µg g^−1^
B	13/120 (10.8%)	Not estimable	—	5/97 (5.15%)	Not estimable	—
Ca	64/120 (53.3%)	168 (102–244)	509	95/97 (97.9%)	325 (179–652)	1.29 × 10^3^
Co	89/120 (74.2%)	0.00632 (0.00340–0.0110)	0.026	74/97 (76.3%)	0.00381 (0.00167–0.00802)	0.040
Fe	120/120 (100%)	7.54 (6.00–9.30)	12.8	97/97 (100%)	5.16 (4.32–6.17)	9.61
Li	16/120 (13.3%)	Not estimable	—	1/97 (1.03%)	Not estimable	—
Mg	111/120 (92.5%)	9.05 (4.85–15.9)	36.6	97/97 (100%)	24.0 (15.7–51.1)	131
Mo	120/120 (100%)	0.0298 (0.0220–0.0370)	0.067	97/97 (100%)	0.0231 (0.0190–0.0290)	0.061
Ni	58/120 (48.3%)	0.0597 (0.0250–0.168)	0.586	55/97 (56.7%)	0.169 (0.0470–0.390)	2.68
Se	120/120 (100%)	0.318 (0.269–0.367)	0.486	97/97 (100%)	0.323 (0.279–0.381)	0.468

Values are presented to a maximum of three significant figures. *n* > LoD = number quantified above the analytical limit of detection. Median (IQR) is shown where distributional estimation was supported; censored medians were estimated using censoring-aware methods, as described in Section 2.6. P95 denotes the 95th percentile. B and Li were highly censored and are therefore reported on a detectability basis rather than through model-dependent central or upper-tail estimates.

**Table 2:** Sex-related comparisons of scalp-hair element distributions in children and adolescents (µg g^−1^).

Element	Boys	Girls	*p*	BH *q*	Male Adolescents	Female Adolescents	*p*	BH *q*
B	5/50 detected	8/70 detected	—	—	1/29 detected	4/68 detected	—	—
Ca	10/50 detected; median not estimable	222 (165–298)	4.90 × 10^−10^	3.92 × 10^−9^	168 (140–616)	375 (247–659)	0.0221	0.0353
Co	0.00474 (0.00306–0.00793)	0.00755 (0.00465–0.0140)	4.45 × 10^−4^	8.90 × 10^−4^	0.00220 (0.000910–0.00336)	0.00528 (0.00296–0.0120)	2.99 × 10^−4^	0.00120
Fe	6.59 (5.23–8.76)	7.86 (6.74–9.60)	0.0171	0.0273	4.52 (3.84–5.32)	5.60 (4.71–6.95)	1.94 × 10^−4^	0.00120
Li	5/50 detected	11/70 detected	—	—	0/29 detected	1/68 detected	—	—
Mg	5.94 (3.23–9.09)	13.4 (7.96–23.2)	1.59 × 10^−7^	6.37 × 10^−7^	16.0 (12.7–94.4)	24.6 (18.5–49.3)	0.123	0.140
Mo	0.031 (0.025–0.036)	0.029 (0.022–0.038)	0.285	0.325	0.021 (0.018–0.027)	0.025 (0.019–0.029)	0.329	0.329
Ni	0.00568 (0.000910–0.0350)	0.117 (0.0540–0.201)	2.99 × 10^−5^	7.98 × 10^−5^	8/29 detected; median not estimable	0.226 (0.0770–0.378)	0.0197	0.0353
Se	0.321 (0.287–0.371)	0.316 (0.262–0.365)	0.526	0.526	0.366 (0.292–0.414)	0.319 (0.273–0.362)	0.0641	0.0854

Values are median (IQR) where stable; otherwise the number detected/number analysed is shown. Censored comparisons used NADA::cendiff, and uncensored comparisons used rank-based tests. The estimability of a subgroup median was evaluated separately from eligibility for a censoring-aware group comparison, thus the 10/50 boys contributing to the Ca comparison and the 8/29 male adolescents contributing to the Ni comparison were retained in the complete censored-distribution tests even though stable subgroup medians were not estimable. B and Li were not subjected to primary sex-related inference because of high censoring or sparse detections. BH *q* = Benjamini–Hochberg false-discovery-rate-adjusted *p*-value.

**Table 3:** Age-group comparisons of scalp-hair element distributions (µg g^−1^).

Element	Children	Adolescents	*p*	BH *q*
B	13/120 detected	5/97 detected	—	—
Ca	168 (102–244)	325 (179–652)	5.36 × 10^−10^	1.43 × 10^−9^
Co	0.00632 (0.00340–0.0110)	0.00381 (0.00167–0.00802)	0.00730	0.00978
Fe	7.54 (6.00–9.30)	5.16 (4.32–6.17)	5.70 × 10^−12^	2.28 × 10^−11^
Li	16/120 detected	1/97 detected	—	—
Mg	9.05 (4.85–15.9)	24.0 (15.7–51.1)	1.11 × 10^−19^	8.91 × 10^−19^
Mo	0.0298 (0.0220–0.0370)	0.0231 (0.0190–0.0290)	4.65 × 10^−6^	9.31 × 10^−6^
Ni	0.0597 (0.0250–0.168)	0.169 (0.0470–0.390)	1.31 × 10^−5^	2.10 × 10^−5^
Se	0.318 (0.269–0.367)	0.323 (0.279–0.381)	0.563	0.563

Values are median (IQR) where stable; otherwise the number detected/number analysed is shown. Censored comparisons used NADA::cendiff, and uncensored comparisons used rank-based tests. B and Li were not subjected to primary age-group inference because of high censoring or sparse detections. BH *q* = Benjamini–Hochberg false-discovery-rate-adjusted *p*-value.

**Table 4:** Residential-zone summaries and overall comparisons of scalp-hair element distributions in adolescents (µg g^−1^).

Element	Zone I	Zone II	Zone III	Zone IV	Overall *p*	BH *q*
Ca	232 (144–290) a	267 (160–596) ab	385 (271–545) b	967 (768–1.24 × 10^3^) c	3.44 × 10^−13^	2.75 × 10^−12^
Co	0.00171 (0.000420–0.00515) a	0.00460 (0.00294–0.00718) b	0.00491 (0.00308–0.00934) b	0.00807 (0.00304–0.0140) b	0.00550	0.0111
Fe	4.83 (4.06–5.83)	5.10 (4.69–5.82)	5.34 (4.25–6.28)	6.17 (4.54–8.15)	0.145	0.185
Mg	18.8 (14.2–28.8) a	19.1 (13.8–31.9) a	24.3 (18.9–35.1) a	113 (94.4–131) b	5.65 × 10^−7^	2.26 × 10^−6^
Mo	0.026 (0.019–0.033) a	0.024 (0.021–0.029) a	0.019 (0.016–0.024) b	0.023 (0.020–0.029) ab	0.00400	0.0106
Ni	0.194 (0.0310–0.552)	9/20 detected; median not estimable	0.162 (0.0580–0.285)	0.267 (0.116–0.390)	0.627	0.627
Se	0.304 (0.236–0.369) a	0.321 (0.289–0.369) ab	0.321 (0.280–0.353) ab	0.378 (0.344–0.399) b	0.0226	0.0361

Values are median (IQR) where stable. Adolescents were represented in Zones I–IV (*n* = 36, 20, 24, and 17, respectively). Censored overall comparisons used NADA::cendiff and uncensored comparisons used Kruskal–Wallis tests. Within each element, zones sharing a lowercase letter did not differ after pairwise Benjamini–Hochberg adjustment; letters are shown only for elements whose overall test remained FDR-significant. B and Li were excluded from primary zone inference because of high censoring. For Ni in Zone II, only 9/20 observations were above the LoD, and a stable subgroup median was not estimable. BH *q* = Benjamini–Hochberg false-discovery-rate-adjusted *p*-value.

## Data Availability

Data for this article are available in Open Science Framework (OSF) at: https://osf.io/69v78/overview?view_only=f05b8ba753324acc9ae8f293231d37a8 (accessed on 15 August 2026).

## Supplementary Materials

The following supporting information can be downloaded at https://www.mdpi.com/article/10.3390/jox16050157/s1, Supplementary Methods S1: Food-frequency questionnaire structure, category regrouping, valid denominators, and statistical reanalysis; Supplementary Methods S2: Analytical QA/QC and certified/reference-material information for the elemental panel; Supplementary Methods S3: Detailed censoring-aware statistical implementation, estimability, and inferential eligibility criteria; Supplementary Methods S4: Statistical audit, reproducibility records, and harmonisation of the child Ni analytical field; Supplementary Methods S5: Soil-data reduction and zone-level hair–soil contextualisation workflow; Table S1: Food-group consumption frequency by age–sex group in children and adolescents; Table S2: Exploratory contrasts in FFQ consumption frequency across age–sex groups using Pearson χ^2^ tests with Benjamini–Hochberg FDR adjustment, by food group; Table S3: Summary of left-censoring percentages (% < LoD) by element, age group, and sex; Table S4: Audit comparison of the legacy substitution-based child nickel field (Ni†) and the final harmonised censoring-aware nickel field (Ni); Table S5: Detailed analytical distribution of scalp-hair elements in children aged 6–9 years from Alcalá de Henares, Spain; Table S6: Detailed analytical distribution of scalp-hair elements in boys aged 6–9 years from Alcalá de Henares, Spain; Table S7: Detailed analytical distribution of scalp-hair elements in girls aged 6–9 years from Alcalá de Henares, Spain; Table S8: Detailed analytical distribution of scalp-hair elements in adolescents aged 13–16 years from Alcalá de Henares, Spain; Table S9: Detailed analytical distribution of scalp-hair elements in male adolescents aged 13–16 years from Alcalá de Henares, Spain; Table S10: Detailed analytical distribution of scalp-hair elements in female adolescents aged 13–16 years from Alcalá de Henares, Spain; Table S11: Detailed residential-zone summaries and overall comparisons of scalp-hair element distributions in children; Table S12: Detailed residential-zone summaries and overall comparisons of scalp-hair element distributions in adolescents; Figure S1: Study area and sampling sites; Figure S2: Food-group consumption frequency distributions across the four age–sex groups; Figure S3: Exploratory hair–hair correlation matrix in adolescents; Figure S4: Primary hair–hair correlation matrix for uncensored elements in the full paediatric cohort; Figure S5: Zone-level ecological correspondence between historical topsoil and paediatric scalp-hair distributions for Fe, Co, Mo, and Ni.
