# Supplementary material for: Paediatric Scalp-Hair Biomonitoring of Nutritionally Relevant and Mixed-Source Elements in Urban–Industrial Spain
Source: J Xenobiot. 2026 Aug 25;16(5):157. doi: 10.3390/jox16050157 (PMC13608820; doi:10.3390/jox16050157)
Supplement: Supplementary file 1 [file jox-16-00157-s001.zip › Supplementary Figures.pdf]

Paediatric Scalp-Hair Biomonitoring of Nutritionally Relevant and Mixed-Source Elements in Urban–Industrial Spain

Antonio Peña-Fernández, M. Ángeles Peña Fernández, Borja Martínez-Alonso, Rafael Moreno-Gómez-Toledano, Tomás Cámara-Pastor, Manuel Higueras and M. Carmen Lobo-Bedmar

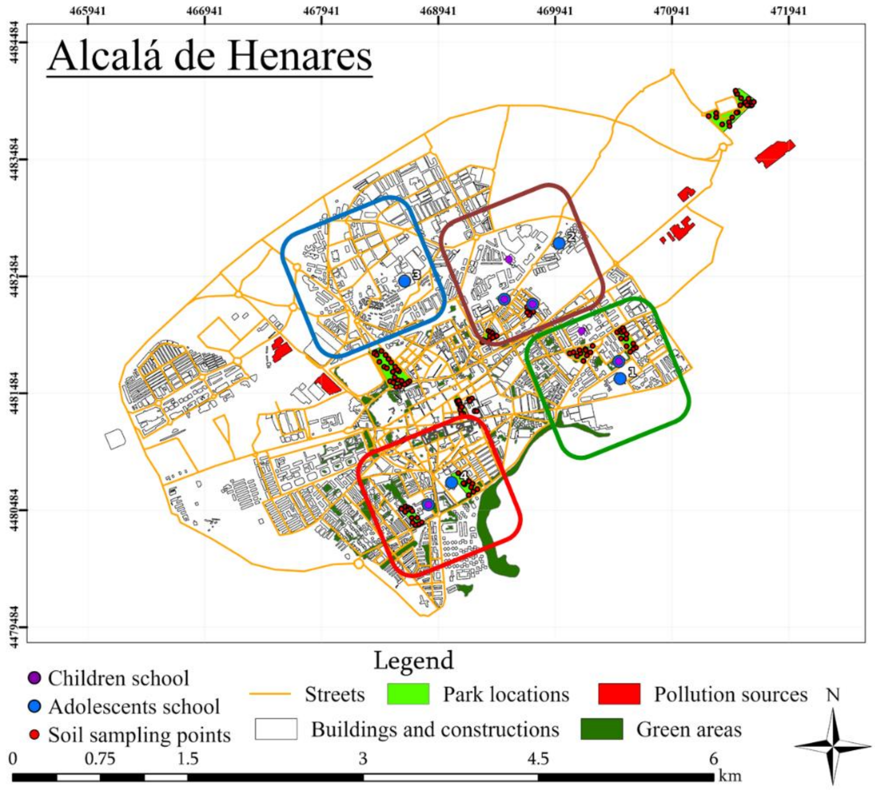

Figure S1. Study area and sampling sites.

Study area, sampling locations, and residential/environmental zones used for the spatial interpretation of paediatric scalp-hair element concentrations in Alcalá de Henares, Spain. Children's primary schools are shown with purple symbols, adolescents' secondary schools with blue symbols, and soil sampling locations with red symbols. Coloured outlines delineate the four residential/environmental zones used to evaluate residential setting: Zone I, lower-density housing with abundant green space; Zone II, compact urban district with dense residential buildings; Zone III, areas characterised by heavy traffic and major roads; and Zone IV, the most heterogeneous land-use setting, combining residential areas with industrial land use and proximity to mapped potential pollution-source locations. Children were recruited from schools located in Zones I, II, and IV, whereas adolescents were enrolled across all four zones. The mapped source locations and zoning are shown for ecological spatial context only and should not be interpreted as participant-level exposure assignments or as evidence of exposure to a specific industrial source. Adapted from the zoning framework used in previous manuscripts from this cohort [23,24].

(A) Food groups, part 1

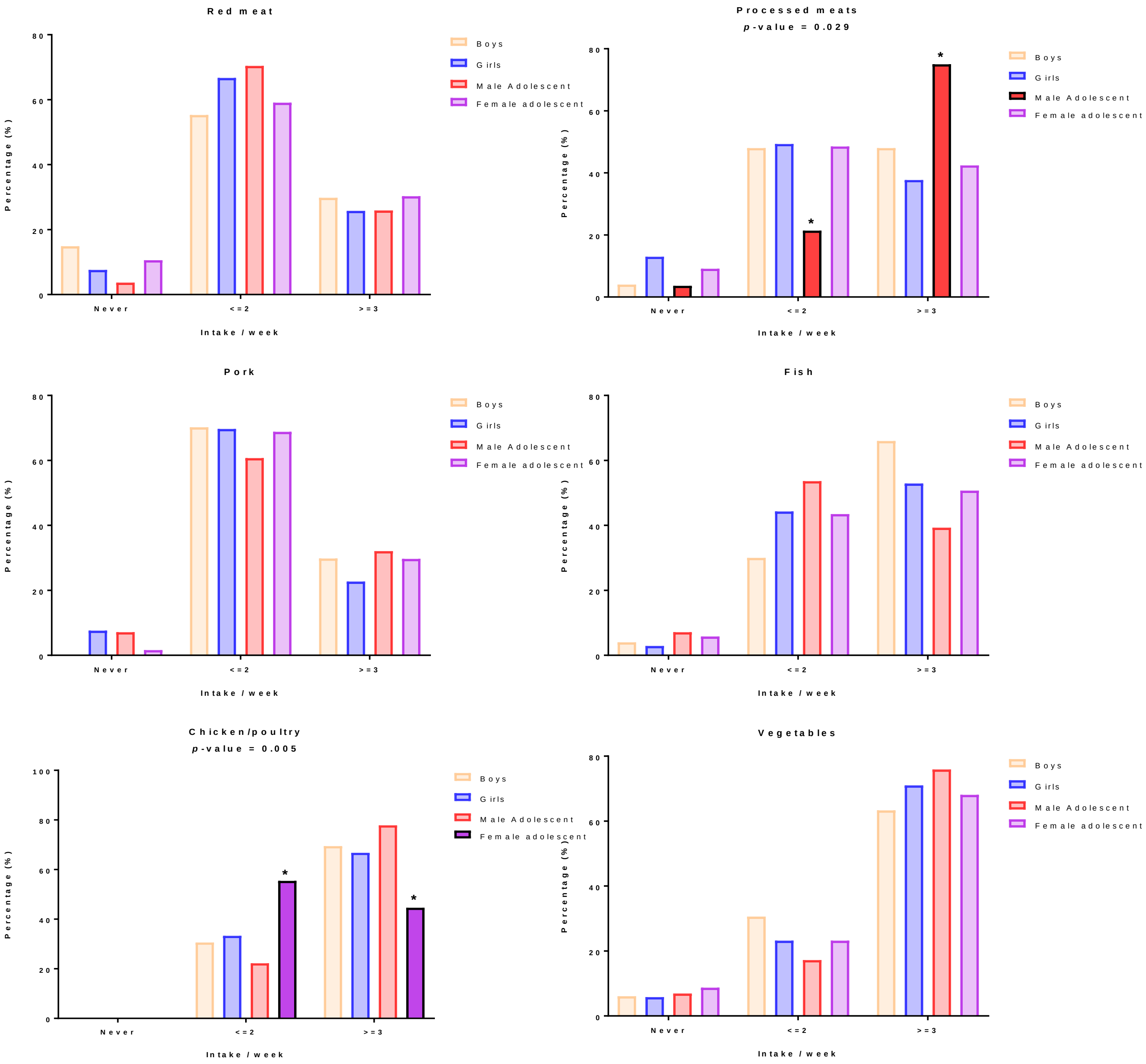

(B) Food groups, part 2

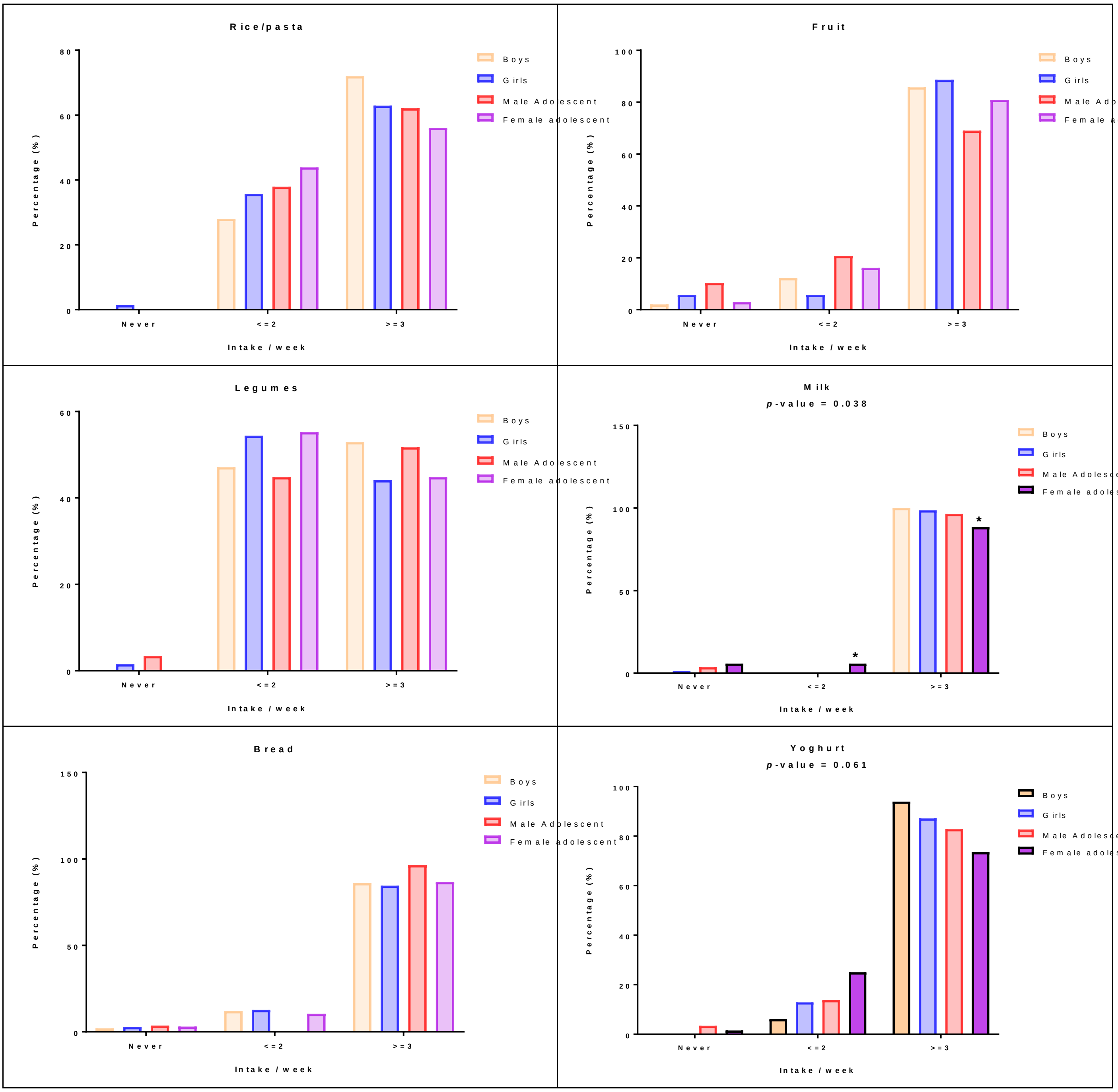

(C) Food groups, part 3

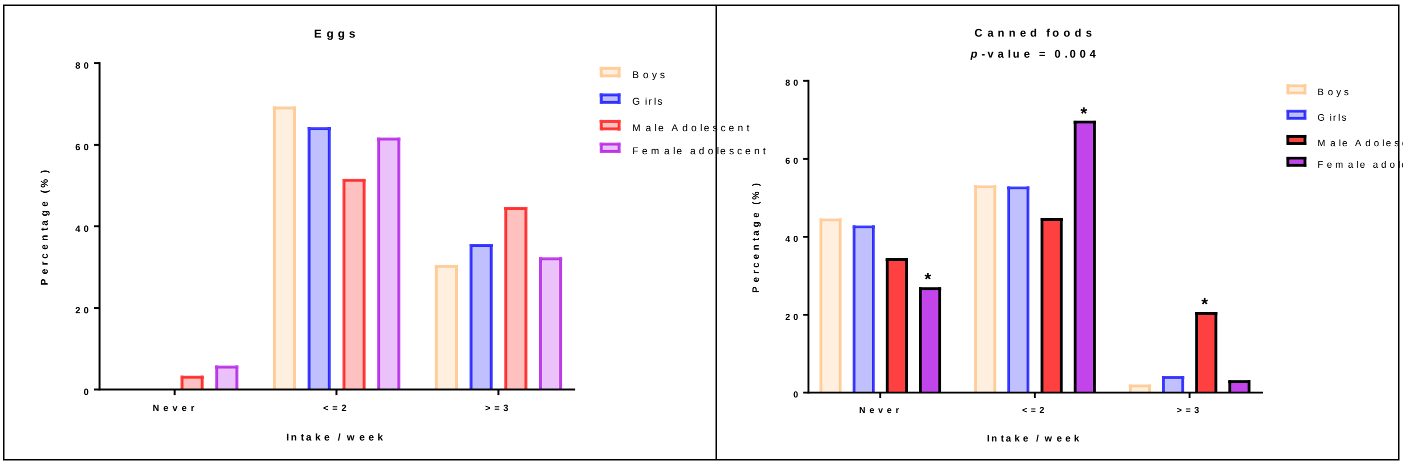

Figure S2. Food-group consumption frequency distributions across the four age–sex groups. Bar plots show the percentage of participants classified as Never, ≤2/week, or ≥3/week for each food group after excluding non-reported or missing responses from the denominator. Groups are shown as boys, girls, male adolescents, and female adolescents. *p*-values correspond to the overall Pearson χ^2 comparison across the four age–sex groups for each food group. After Benjamini–Hochberg correction across the 14 food groups, only chicken/poultry and canned foods remained statistically significant; processed meats and milk were considered nominal exploratory dietary-context signals. Asterisks shown within individual panels denote cells with notable adjusted residuals contributing to the overall χ^2 pattern and should not be interpreted as FDR-adjusted food-group significance.

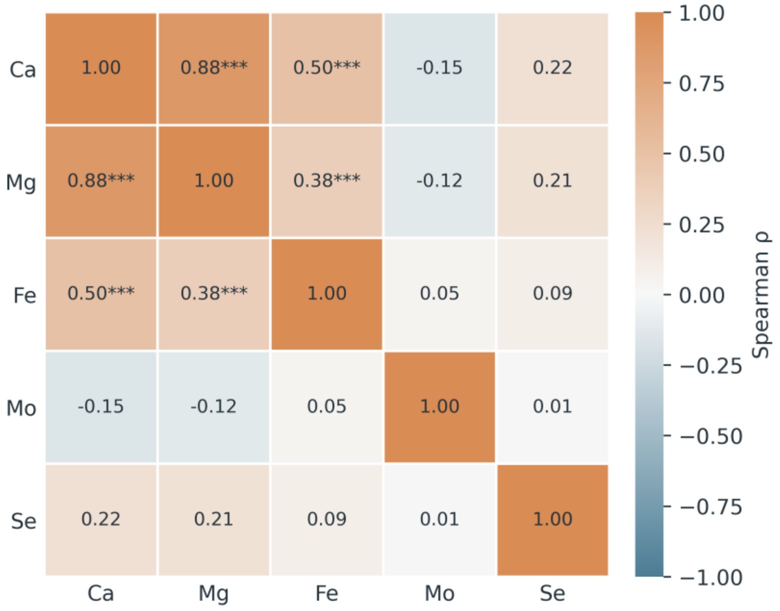

Figure S3. Exploratory hair–hair correlation matrix in adolescents. The heatmap shows pairwise Spearman rank correlation coefficients among Ca, Mg, Fe, Mo, and Se (n = 97). Mg, Fe, Mo, and Se were uncensored, whereas Ca had 2.1% censoring; LoD-bounded Ca observations were retained with their induced rank ties. Benjamini–Hochberg adjustment was applied within the matrix; ***q < 0.001. Because Ca included a small censored fraction, this matrix is classified as exploratory and should not be interpreted as evidence of physiological coupling, shared exposure source, or causality.

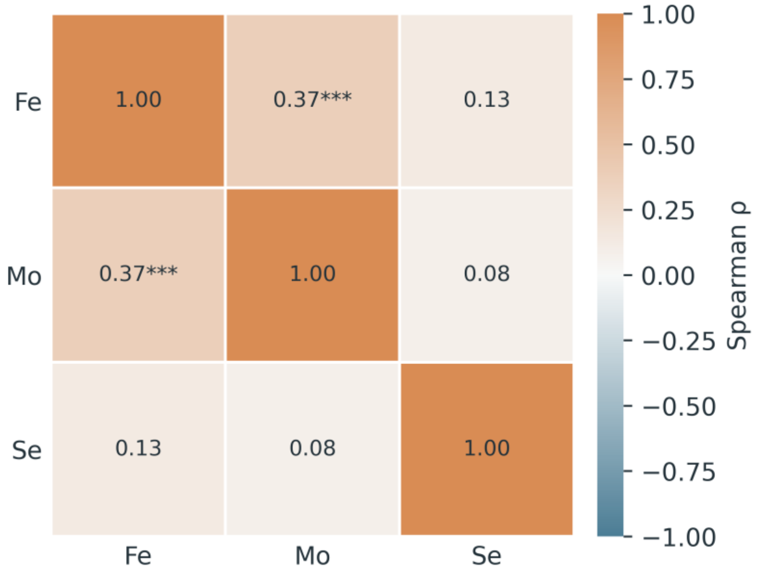

Figure S4. Primary hair–hair correlation matrix for uncensored elements in the full paediatric cohort. The heatmap shows pairwise Spearman rank correlation coefficients for Fe, Mo, and Se (n = 217), the only elements quantified in all participants in both age strata. Benjamini–Hochberg adjustment was applied to the three pairwise correlations; ***$q < 0.001$. Correlations are interpreted as co-occurrence signals and not as evidence of a common source, physiological coupling, or causality.

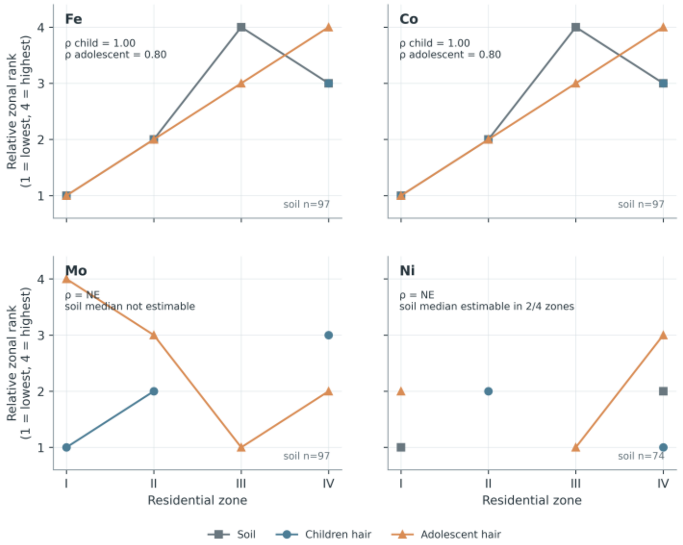

Figure S5. Zone-level ecological correspondence between historical topsoil and paediatric scalp-hair distributions for Fe, Co, Mo, and Ni. Each panel shows the relative zonal rank of the zone-specific median (1 = lowest; progressively higher ranks = higher zonal medians; the maximum rank depended on the number of estimable zones) for independent soil locations, child hair, and adolescent hair across the zones for which a median was estimable. Soil observations were reduced to independent sampling locations before zonal summarisation (n = 97 for Fe, Co, and Mo; n = 74 for Ni). Children were represented in Zones I, II, and IV, whereas adolescents were represented in Zones I–IV. Descriptive Spearman rank concordance was ρ = 1.00 for children and ρ = 0.80 for adolescents for both Fe and Co. Mo correspondence was not estimable because soil censoring ranged

from 87.5% to 100%, and Ni medians were simultaneously estimable in too few zones. No participant-level soil values were assigned and no inferential *p*-values were calculated.
