# Supplementary material for: Paediatric Scalp-Hair Biomonitoring of Nutritionally Relevant and Mixed-Source Elements in Urban–Industrial Spain
Source: J Xenobiot. 2026 Aug 25;16(5):157. doi: 10.3390/jox16050157 (PMC13608820; doi:10.3390/jox16050157)
Supplement: Supplementary file 1 [file jox-16-00157-s001.zip › Supplementary Methods.pdf]

Paediatric Scalp-Hair Biomonitoring of Nutritionally Relevant and Mixed-Source Elements in Urban–Industrial Spain

Antonio Peña-Fernández, M. Ángeles Peña Fernández , Borja Martínez-Alonso, Rafael Moreno-Gómez-Toledano, Tomás Cámara-Pastor, Manuel Higueras and M. Carmen Lobo-Bedmar

Supplementary Methods S1. Food-Frequency Questionnaire Structure, Category Regrouping, Valid Denominators, and Statistical Reanalysis

Habitual food-consumption frequency was assessed during the original 2001 paediatric biomonitoring programme using a brief food-group food-frequency questionnaire (FFQ) consistent with Spanish paediatric dietary-research traditions and food-frequency approaches used in the enKid study and subsequent dietary studies in children and adolescents [28–31]. The questionnaire covered 14 major food groups: red meat, pork, chicken/poultry, processed meats (cured meats/sausages), fish, vegetables, rice/pasta, legumes, bread, fruit, milk, yoghurt, eggs, and canned foods.

Original responses were recorded using the ordered categories Never, <2 times/week, 3–4 times/week, and ≥5 times/week, with non-reported responses coded separately. For the present retrospective reanalysis, these categories were harmonised into three common frequency groups: Never, ≤2 times/week, and ≥3 times/week. The ≥3 times/week category combined the original 3–4 times/week and ≥5 times/week categories and was used as a descriptive indicator of relatively frequent consumption. Regrouping was performed at the level of the archived aggregate frequency tables rather than from participant-level dietary records.

Non-reported or missing responses were excluded separately from the denominator for each food group. Consequently, valid denominators varied slightly among food items and did not necessarily correspond exactly to the sample sizes of the associated hair-analytical strata. These differences reflect the structure of the historical aggregated FFQ outputs, category regrouping, and item-specific non-reporting and do not indicate participant duplication. Participant-level dietary records, quantitative portion sizes, total dietary intake, supplement use, food-composition linkage, drinking-water intake, and residential-zone-specific dietary data were not available.

Consumption-frequency distributions were evaluated across four age–sex strata: boys aged 6–9 years, girls aged 6–9 years, male adolescents aged 13–16 years, and female adolescents aged 13–16 years. For each food group, results were expressed as counts and row percentages among participants with valid information. Pearson χ^2 tests were used to evaluate overall heterogeneity in the collapsed consumption-frequency distributions across the four age–sex groups. Adjusted residuals from each contingency table were retained as descriptive indicators of the individual cells contributing most strongly to the overall χ^2 statistic. For chicken/poultry, no participant was classified in the Never category; the statistical comparison therefore involved the two observed categories, ≤2/week and ≥3/week, while the zero-frequency Never category was retained in the descriptive presentation for consistency across food groups.

Because 14 food-group comparisons were performed, raw p-values were adjusted using the Benjamini–Hochberg false-discovery-rate procedure [32]. The complete age–sex distributions, valid denominators, Pearson χ^2 statistics, degrees of freedom, adjusted residuals, and unadjusted p-values are reported in Supplementary Table S1. For additional developmental-stage description, boys and girls were pooled to derive overall child frequencies and male and female adolescents were pooled to derive overall adolescent frequencies. The percentages consuming each food group ≥3 times/week and the Benjamini–Hochberg-adjusted results are reported in Supplementary Table S2. In the updated reanalysis, only chicken/poultry and canned-food consumption remained FDR-robust (both $q = 0.035$); processed meats and milk showed nominal differences before adjustment but did not remain significant after FDR correction.

The corresponding graphical presentation is provided in Supplementary Figure S2. Importantly, the FFQ data were retained exclusively as population-level dietary context. They were not used to estimate nutrient or elemental

intake, assess dietary adequacy, or model food consumption as an individual-level determinant of scalp-hair elemental concentrations. The same historical FFQ source had been considered in previous work from this cohort [24]; however, Supplementary Tables S1 and S2 and Supplementary Figure S2 report the updated reanalysis used in the present manuscript, including revised valid denominators, harmonised frequency categories, Pearson χ^2 statistics, adjusted residuals, and Benjamini–Hochberg-adjusted q-values.

Supplementary Methods S2. Analytical QA/QC and Certified/Reference-Material Information for the Elemental Panel

Analytical quality assurance and quality control (QA/QC) were implemented throughout sample preparation and ICP–MS determination and included procedural digestion blanks, calibration-verification standards, duplicate analyses, replicate instrumental readings, continuous internal-standard monitoring, and analysis of a human-hair reference material. The overall analytical strategy was consistent with that used in previous investigations of archived hair from the same paediatric cohort [23,24]. Because scalp hair can retain externally deposited particulate material and the effectiveness of decontamination is an important determinant of elemental hair analysis, all study samples and reference-material aliquots were handled within the same quality-controlled analytical framework described in the main manuscript [26,33].

NCS DC 73347 human-hair certified reference material (National Research Centre for Certified Reference Materials, Beijing, China) was used as the principal keratin-matrix quality-control material and was analysed periodically throughout the analytical sequence, approximately every five study samples. For the elemental panel evaluated in the present investigation, the assigned certified or reference concentrations were 2.00 μg g^{-1} for Li, 1.30 μg g^{-1} for B, 2.90 × 10^3 μg g^{-1} for Ca, 0.0710 μg g^{-1} for Co, 54.0 μg g^{-1} for Fe, 360 μg g^{-1} for Mg, 0.0730 μg g^{-1} for Mo, 0.830 μg g^{-1} for Ni, and 0.600 μg g^{-1} for Se. The manufacturer reports the B concentration as an uncertified reference value rather than a certified value; this distinction was retained throughout QA/QC interpretation.

The concentrations obtained for NCS DC 73347 were 1.83 μg g^{-1} for Li, 1.41 μg g^{-1} for B, 2.80 × 10^3 μg g^{-1} for Ca, 0.0655 μg g^{-1} for Co, 55.7 μg g^{-1} for Fe, 350 μg g^{-1} for Mg, 0.0680 μg g^{-1} for Mo, 0.818 μg g^{-1} for Ni, and 0.556 μg g^{-1} for Se. Relative to the corresponding certified or reference concentrations, analytical recoveries were 91.3% for Li, 109% for B, 96.6% for Ca, 92.2% for Co, 103% for Fe, 97.2% for Mg, 93.2% for Mo, 98.6% for Ni, and 92.6% for Se. For the certified constituents, recoveries therefore ranged from 91.3% to 103%; the 109% value for B was calculated against its manufacturer-reported uncertified reference concentration. These element-specific results provide the quantitative CRM performance information underlying the abbreviated QA/QC description in the main manuscript.

Procedural digestion blanks were included at a frequency of approximately one blank per five study samples to monitor contamination introduced during sample handling, washing, digestion, and subsequent preparation. Calibration-verification standards and procedural blanks were measured at the beginning and end of each analytical sequence. Niobium was continuously introduced as an internal standard to monitor instrumental drift and matrix-related signal variability, while each digest was analysed in duplicate with three replicate instrumental readings per sample. The monitored isotopes were ^{11}B, ^{44}Ca, ^{59}Co, ^{56}Fe, ^{7}Li, ^{24}Mg, ^{97}Mo, ^{60}Ni, and ^{77}Se.

Element-specific analytical limits of detection (LoDs) were calculated as three times the standard deviation of the procedural-blank signal (3σ). LoDs were derived separately for the child and adolescent analytical datasets. Small differences between age strata reflected differences in available post-washing hair mass and associated dilution requirements rather than changes in ICP–MS performance. Accordingly, detection frequency and censoring were evaluated against the age-stratum-specific analytical LoD throughout the statistical analysis.

The CRM data were used to assess analytical accuracy within a keratinised matrix, whereas procedural blanks, calibration-verification standards, replicate measurements, and internal-standard behaviour provided complementary information on contamination control, calibration stability, repeatability, and instrumental performance. The resulting QA/QC framework supports the internal comparability of the archived child and adolescent datasets but does not

remove the broader interpretive limitations inherent to scalp-hair biomonitoring, including potential residual exogenous contribution after washing [25,26,33].

Supplementary Methods S3. Detailed Censoring-Aware Statistical Implementation, Estimability, and Inferential Eligibility Criteria

Analytical observations reported below the element-specific limit of detection (LoD) were represented explicitly as left-censored observations. The analytical LoD defined the upper bound of the censored observation and was not treated as an observed concentration. No replacement by zero, LoD/2, LoD/√2, or another fixed substitution value was used for primary descriptive estimation or inference, consistent with established recommendations for environmental and biomonitoring datasets containing nondetects [34–36,40].

For uncensored variables, descriptive statistics were calculated empirically from the observed concentrations. For variables with low or moderate censoring and sufficient detectable information, reverse Kaplan–Meier estimation was implemented using NADA::cenfit(). Where the distribution contained sufficient information for modelling but the relevant Kaplan–Meier quantiles were not estimable, regression on order statistics (ROS) was examined using NADA::cenros() to obtain central distributional descriptors. NADA2::cfit() was used as an implementation-consistency check where applicable. Model-derived summaries were interpreted as estimates of censored distributions rather than as directly observed concentrations [34–36].

Analytical strata with extensive censoring or insufficient detections to support stable distributional estimation were characterised principally by sample size, number and percentage of observations above and below the LoD, the analytical LoD, and the detectable portion of the concentration range. A prespecified high-censoring threshold of ≥80% was used as a general boundary above which primary model-dependent distributional estimation and inferential testing were not undertaken. Below this threshold, the availability and distribution of detected observations were additionally considered before determining whether a descriptive estimate or censoring-aware comparison was sufficiently supported. Highly censored analytes, particularly B and Li, were therefore interpreted principally through detectability rather than unstable estimates of central tendency or upper-tail behaviour.

Where the available distributional information supported their estimation, upper-tail descriptors included the 95th percentile (P95) and 97.5th percentile (P97.5). Confidence intervals for P95 were estimated using a 20,000-replicate nonparametric bootstrap of participant-level records. These percentiles were retained exclusively as historical population-level screening descriptors and were not interpreted as clinical, nutritional, or toxicological thresholds.

For comparisons involving censored variables with sufficient analytical information, between-group differences were evaluated using NADA::cendiff(), implementing a Peto–Peto/Gehan–Wilcoxon-type comparison of censored empirical distributions as a G-rho test with rho = 1 [41,42]. NADA2::cen1way() was used as an independent implementation check where applicable. Uncensored two-group comparisons used Wilcoxon rank-sum tests, whereas uncensored comparisons involving more than two groups used Kruskal–Wallis tests followed, where appropriate, by pairwise Wilcoxon rank-sum comparisons. Residential-zone pairwise comparisons followed the corresponding censoring-aware or uncensored framework [34–36].

Importantly, estimability of a descriptive statistic within an individual subgroup was assessed separately from eligibility of the complete comparison dataset for censoring-aware inference. Thus, a subgroup containing too few detections to support a stable median could nevertheless contribute all of its censored and uncensored observations to an overall cendiff() comparison when the comparison dataset as a whole contained sufficient detectable information. Conversely, primary inferential testing was not performed when the complete comparison dataset showed ≥80% censoring or otherwise lacked sufficient detectable information to support a reliable comparison.

Raw p-values were adjusted within the relevant prespecified families of element-wise comparisons using the Benjamini–Hochberg false-discovery-rate procedure [32]. Adjusted q-values <0.05 were considered FDR-robust, while nominal associations that did not remain significant after adjustment were retained only as exploratory signals.

Interpretation additionally considered censoring intensity, detection frequency, effect direction, and distributional consistency.

All analyses were conducted in R [43]. Censored-data estimation and inference principally used NADA version 1.6-1.1 and NADA2 version 1.1.6 [36]. Package versions, analytical decision records, implementation checks, and run-level quality-control information were retained as part of the reproducibility archive. The separate record-linkage and harmonisation audit for the child Ni analytical field is described in Supplementary Methods S4.

Supplementary Methods S4. Statistical Audit, Reproducibility Records, and Harmonisation of the Child Ni Analytical Field

A dedicated record-linkage and analytical audit was conducted for the child Ni dataset to ensure that the final censoring-aware analytical field was reconstructed directly from the analytical source rather than from previously derived or substitution-based variables. The procedure was implemented in R [43], with data import, key validation, record linkage, censoring reconstruction, and quality-control outputs retained as part of the reproducibility archive.

The primary linkage variable was the individual analytical sample reference recorded in the source files as Referencia. For computational matching, this field was internally normalised to generate participant_ref_key by standardising case, removing diacritical marks, trimming whitespace, and removing non-alphanumeric characters. This normalisation was used solely to standardise the representation of the identifier; the linkage itself remained an exact reference-based match and did not involve fuzzy matching, probabilistic record linkage, row-position matching, or matching on participant characteristics. Both the principal child dataset and the analytical Ni/V source contained 120 records and 120 unique analytical references.

Before linkage, the workflow verified the absence of missing Referencia values and confirmed uniqueness of the identifier in both source datasets. Bidirectional anti_join() checks were then performed to identify references occurring in only one source. No unmatched references were identified in either direction. The subsequent reference-based linkage yielded 120/120 successfully linked records for Ni. As an independent source-file integrity check, the co-located V field also yielded 120/120 linked records; V was not otherwise included in the present study. No duplicate analytical references were retained.

Participant name (Nombre), sex/gender (Género), and residential zone (Zonas) were used exclusively as secondary post-link validation fields. They were not used to determine the primary match. This distinction was important because the archived participant information contained a duplicated name, whereas Referencia remained unique. Post-link comparison identified zero discrepancies in participant name, zero discrepancies in sex, and zero discrepancies in residential zone across the 120 linked records. Variables such as school (Colegio) and sample mass (Gramos) were not used to determine record linkage. The audit script was configured to stop and produce a discrepancy output if unmatched references, duplicated identifiers, or disagreements in the secondary validation fields were detected.

For harmonisation of Ni, the workflow explicitly located the final analytical concentration block expressed in $\mu g\ g^{-1}$ and its corresponding element-specific LoD within the archived analytical source file. This step was necessary because the workbook contained repeated analytical blocks generated at different stages of data processing, and the workflow was designed to prevent inadvertent extraction of an earlier intermediate field. The final Ni concentration was extracted directly from this analytical block together with its corresponding analytical limit of detection.

The final LoD for child Ni was 0.020 $\mu g\ g^{-1}$. The original analytical value was retained separately as Ni_source_value, and an explicit censoring indicator was generated for observations at or below the LoD. For construction of the censored-data object, observations with analytical values ≤LoD were represented by the LoD as the censoring limit (Ni_cen_value) together with the corresponding censoring flag. Importantly, the LoD value was used only to define the upper bound of the left-censored observation and was not treated as an observed concentration or as

a substitution value for statistical inference. No replacement by zero, LoD/2, LoD/√2, or another fixed value was used in the primary analysis, consistent with established recommendations for the analysis of nondetects [34–36,40].

The harmonised analytical field was subsequently designated Ni in the final analysis dataset, while Ni_LoD and Ni_censored were retained in parallel to preserve the analytical limit and censoring status of each observation. This structure maintained traceability from the source analytical value through censoring classification to the final statistical representation and allowed the censored and uncensored observations to be analysed jointly using the censoring-aware procedures described in the main manuscript.

The historical substitution-based field designated Ni† was not used to reconstruct, calibrate, modify, or otherwise derive the final Ni field. It was retained exclusively as a legacy/provenance variable for methodological audit and comparison with the historical treatment of the dataset [39]. The workflow explicitly designated the harmonised Ni field as the canonical variable for primary inference, while historical, manually derived, or substitution-based Ni fields were classified as excluded from primary inference. Consequently, there was no mathematical conversion or calibration from Ni† to Ni: the final Ni field originated independently from the analytical source concentration, its LoD, and the corresponding censoring indicator. The numerical consequences of the two approaches are reported in Supplementary Table S4.

Data import and record-linkage auditing principally used readxl::read_excel(), dplyr::anti_join(), dplyr::left_join(), and base-R functions including iconv(), tolower(), gsub(), trimws(), duplicated(), and anyDuplicated(). Quality-control workbooks were exported using writexl::write_xlsx(). No fuzzy-matching or probabilistic record-linkage package was used. Subsequent descriptive estimation and inference for the harmonised censored Ni field followed the general censoring-aware statistical framework, distributional estimability criteria, and inferential eligibility rules described in Supplementary Methods S3. No separate Ni-specific statistical model, censoring threshold, or inferential criterion was applied.

Additional reproducibility safeguards were implemented throughout the workflow. The analytical source value, LoD, and censoring flag were retained as separate fields so that every censored observation remained traceable to its analytical origin. Final-run logs recorded the input files, record counts, detected analytical fields, output status, and relevant software information. Expected-output checks were completed at the end of the workflow. Together, these procedures confirmed complete and internally consistent linkage of the child analytical dataset and ensured that all primary Ni results reported in the present study derive from the harmonised analytical source rather than from the historical substitution-based field.

Supplementary Methods S5. Soil-Data Reduction and Zone-Level Hair–Soil Contextualisation Workflow

Historical topsoil data from Alcalá de Henares were used exclusively as secondary ecological context for the scalp-hair biomonitoring results. The environmental dataset originated from the previously published investigation of historical urban, industrial, and garden topsoils from Alcalá de Henares [22]. The present hair–soil contextualisation was restricted to elements represented analytically in both datasets and for which sufficient information was available to support meaningful ecological comparison. The overlapping analytical panel comprised Co, Fe, Mo, and Ni. Ca, Mg, B, and Li were not included because corresponding measurements were not available in the overlapping local soil panel used for this analysis. Se was excluded because it was not detected in the local topsoil dataset at the reported analytical limit.

The hair and soil datasets differed fundamentally in sampling design and were therefore not treated as individually paired datasets. Hair observations represented individual paediatric participants, whereas the environmental dataset contained soil analytical observations arising from independent sampling locations together with analytical replication. Before any zonal comparison, the soil data were reduced to the independent sampling-location or composite level so that analytical replication did not artificially increase the environmental sample size.

After this reduction, the contextual soil dataset comprised 97 independent locations for Co, Fe, and Mo and 74 independent locations for Ni, with no duplicated analytical rows retained within the independent-location datasets.

Residential-zone assignment followed the same four-zone spatial framework used for the paediatric cohort and previous investigations from this study population [23,24]. Children were represented in Zones I, II, and IV, whereas adolescents were represented in Zones I–IV. Hair and soil data were summarised separately within each corresponding residential/environmental zone; soil concentrations were never copied or assigned to individual participant records. This approach was adopted specifically to avoid pseudoreplication and to preserve the ecological level of the environmental comparison.

For each element and dataset, zone-specific distributions were characterised using medians and interquartile ranges where these statistics were estimable. Where censoring prevented stable estimation of a zonal median or other distributional descriptors, the available information was represented by detection frequency, percentage censored, and the defensible estimable portion of the distribution. For graphical comparison across hair and soil datasets measured on different concentration scales, estimable zonal medians were converted to within-dataset ordinal ranks, with rank 1 representing the lowest zonal median and progressively higher ranks representing higher zonal medians; the maximum attainable rank therefore depended on the number of zones for which a median was estimable. Supplementary Figure S5 presents these rank-based ecological comparisons for Fe, Co, Mo, and Ni. The underlying soil summaries were derived from independent sampling locations ($n = 97$ for Fe, Co, and Mo; $n = 74$ for Ni).

Where a sufficient number of corresponding zone-level medians was available, Spearman's rank correlation coefficient (ρ) was calculated descriptively between the ordering of hair and soil zones. Separate coefficients were considered for the three child zones and the four adolescent zones. These calculations were ecological rank-concordance descriptors only; no inferential p-values were calculated, because the effective sample size was only three or four zones and conventional significance testing would not provide a meaningful basis for participant-level inference. No soil median or mean was replicated across individual hair records, and no participant-level hair–soil correlation was performed. This distinction was maintained to avoid both pseudoreplication and inappropriate attribution of an ecological environmental measure to individual participants.

For Fe, the relative zonal ordering of soil and hair medians was identical across the three child zones, producing a descriptive Spearman coefficient of $\rho = 1.00$, and strong concordance was observed across the four adolescent zones ($\varrho = 0.800$). Co yielded the same descriptive coefficients. A comparable rank coefficient was not estimable for Mo because soil censoring ranged from 87.5% to 100% across zones, preventing stable estimation of the required soil medians. For Ni, hair and soil medians were simultaneously estimable in too few corresponding zones to support a meaningful rank coefficient. These estimability constraints are retained explicitly rather than being resolved through substitution or imputation.

The hair–soil analysis was therefore designed to answer a deliberately limited question: whether broad residential-zone ordering in paediatric hair was compatible with the zonal ordering observed in historical local topsoil for overlapping elements. It was not designed to estimate uptake, bioavailability, individual exposure, source contribution, or direct soil-to-hair transfer. The observed Fe and Co concordance should consequently be interpreted only as ecological compatibility between the two historical datasets. The approach does not distinguish ingestion, inhalation, dermal contact, or external deposition and cannot establish causal environmental attribution [22,25,26].

References cited in Supplementary Methods

Reference numbering corresponds to that used in the main manuscript.

22. Peña-Fernández, A.; Higueras, M.; Tepanosyan, G.; Peña Fernández, M.Á.; Lobo, M.C. Geochemical patterns and human health risks of less-regulated metal(loid)s in historical urban and industrial topsoils from Alcalá de Henares, Spain. *J. Xenobiot.* **2026**, *16*, 17. https://doi.org/10.3390/jox16010017.

23. Peña-Fernández, A.; Higueras, M.; Valiente Borox, R.; Lobo-Bedmar, M.C. Rare earth elements and technology-related trace metals in paediatric scalp hair: A 2001 urban baseline from Spain. *J. Xenobiot.* **2026**, *16*, 38. https://doi.org/10.3390/jox16010038.

24. Peña-Fernández, A.; Valiente, R.; Higueras, M.; Moreno Gómez-Toledano, R.; Lobo-Bedmar, M.C. Ba–Sr–V as geogenic and traffic tracers in paediatric hair from urban–industrial Spain, with co-located topsoil vanadium. *Toxics* **2026**, *14*, 268. https://doi.org/10.3390/toxics14030268.

25. Rodrigues, J.L.; Batista, B.L.; Nunes, J.A.; Passos, C.J.; Barbosa, F., Jr. Evaluation of the use of human hair for biomonitoring the deficiency of essential and exposure to toxic elements. *Sci. Total Environ.* **2008**, *405*, 370–376. https://doi.org/10.1016/j.scitotenv.2008.06.002.

26. Pozebon, D.; Scheffler, G.L.; Dressler, V.L. Elemental hair analysis: A review of procedures and applications. *Anal. Chim. Acta* **2017**, *992*, 1–23. https://doi.org/10.1016/j.aca.2017.09.017.

28. Aranceta, J.; Pérez-Rodrigo, C.; Ribas, L.; Serra-Majem, L. Sociodemographic and lifestyle determinants of food patterns in Spanish children and adolescents: The enKid study. *Eur. J. Clin. Nutr.* **2003**, *57*, S40–S44. https://doi.org/10.1038/sj.ejcn.1601813.

29. Larroya, A.; Tamayo, M.; Cenit, M.C.; Sanz, Y. Validity and reproducibility of a Spanish EPIC food frequency questionnaire in children and adolescents. *Nutrients* **2024**, *16*, 3809. https://doi.org/10.3390/nu16223809.

30. Serra-Majem, L.; García-Closas, R.; Ribas, L.; Pérez-Rodrigo, C.; Aranceta, J. Food patterns of Spanish schoolchildren and adolescents: The enKid Study. *Public Health Nutr.* **2001**, *4*, 1433–1438. https://doi.org/10.1079/PHN2001234.

31. Serra-Majem, L.; Ribas, L.; Ngo, J.; Ortega, R.M.; García, A.; Pérez-Rodrigo, C.; Aranceta, J. Food, youth and the Mediterranean diet in Spain. Development of KIDMED, Mediterranean Diet Quality Index in children and adolescents. *Public Health Nutr.* **2004**, *7*, 931–935. https://doi.org/10.1079/PHN2004556.

32. Benjamini, Y.; Hochberg, Y. Controlling the false discovery rate: A practical and powerful approach to multiple testing. *J. R. Stat. Soc. Ser. B* **1995**, *57*, 289–300. https://doi.org/10.1111/j.2517-6161.1995.tb02031.x.

33. Schuhmacher, M.; Bellés, M.; Rico, A.; Domingo, J.L.; Corbella, J. Impact of reduction of lead in gasoline on the blood and hair lead levels in the population of Tarragona Province, Spain, 1990–1995. *Sci. Total Environ.* **1996**, *184*, 203–209. https://doi.org/10.1016/0048-9697(96)05102-9.

34. Helsel, D.R. *Statistics for Censored Environmental Data Using Minitab and R*, 2nd ed.; John Wiley & Sons: Hoboken, NJ, USA, 2011.

35. Shoari, N.; Dubé, J.S. Toward improved analysis of concentration data: Embracing nondetects. *Environ. Toxicol. Chem.* **2018**, *37*, 643–656. https://doi.org/10.1002/etc.4046.

36. Julian, P.; Helsel, D.; Lee, L. NADA2: Data Analysis for Censored Environmental Data, R Package Version 1.1.6. 2024. Available online: https://CRAN.R-project.org/src/contrib/Archive/NADA2/ (accessed on 3 July 2026).

39. Peña-Fernández, A. Presencia y Distribución Medioambiental de Metales Pesados y Metaloides en Alcalá de Henares, Madrid. Evaluación del Riesgo Para la Población y Biomonitorización de la Población Escolar. Ph.D. Thesis, Universidad de Alcalá, Alcalá de Henares, Spain, 2011. Available online: https://ebuah.uah.es/xmlui/handle/10017/9510 (accessed on 15 August 2026).

40. Hladik, M.L.; Markus, A.; Helsel, D.; Nowell, L.H.; Polesello, S.; Rüdel, H.; Szabo, D.; Wilson, I. Evaluating the reliability of environmental concentration data to characterize exposure in environmental risk assessments. *Integr. Environ. Assess. Manag.* **2024**, *20*, 981–1003. https://doi.org/10.1002/ieam.4893.

41. Peto, R.; Peto, J. Asymptotically efficient rank invariant test procedures. *J. R. Stat. Soc. Ser. A* **1972**, *135*, 185–198. https://doi.org/10.2307/2344317.

42. Klein, J.P.; Moeschberger, M.L. *Survival Analysis: Techniques for Censored and Truncated Data*, 2nd ed.; Springer: New York, NY, USA, 2003. https://doi.org/10.1007/b97377.

43. R Core Team. *R: A Language and Environment for Statistical Computing*; R Foundation for Statistical Computing: Vienna, Austria, 2026. Available online: https://www.R-project.org/ (accessed on 3 July 2026).
