# Supplementary material for: Paediatric Scalp-Hair Biomonitoring of Nutritionally Relevant and Mixed-Source Elements in Urban–Industrial Spain
Source: J Xenobiot. 2026 Aug 25;16(5):157. doi: 10.3390/jox16050157 (PMC13608820; doi:10.3390/jox16050157)
Supplement: Supplementary file 1 [file jox-16-00157-s001.zip › Supplementary Tables.pdf]

Paediatric Scalp-Hair Biomonitoring of Nutritionally Relevant and Mixed-Source Elements in Urban–Industrial Spain

Antonio Peña-Fernández, M. Ángeles Peña Fernández , Borja Martínez-Alonso, Rafael Moreno-Gómez-Toledano, Tomás Cámara-Pastor, Manuel Higueras and M. Carmen Lobo-Bedmar

Table S1. Food-group consumption frequency by age–sex group in children and adolescents.

Food group	Population group	Sex	Statistic	Never (%)	≤ 2/week (%)	≥ 3/week (%)	Total (excl. NR, %)	Pearson χ^2	df	*p*-value
Red meat	Children	Boys	Count	7	26	14	47	3.966	6	0.681
			% within population group	14.9%	55.3%	29.8%	100.0%			
			Adjusted residual	1.4	-1.1	0.3				
		Girls	Count	5	44	17	66			
			% within population group	7.6%	66.7%	25.8%	100.0%			
			Adjusted residual	-0.7	0.9	-0.5				
	Adolescents	Male	Count	1	19	7	27			
			% within population group	3.7%	70.4%	25.9%	100.0%			
			Adjusted residual	-1.1	0.9	-0.3				
		Female	Count	7	39	20	66			
			% within population group	10.6%	59.1%	30.3%	100.0%			
			Adjusted residual	0.3	-0.6	0.5				
	Total		Count	20	128	58	206			
			% within population group	9.7%	62.1%	28.2%	100.0%			

Food group	Population group	Sex	Statistic	Never (%)	≤ 2/week (%)	≥ 3/week (%)	Total (excl. NR, %)	Pearson χ^2	df	*p*-value
Pork	Children	Boys	Count	0	33	14	47	6.999	6	0.321
			% within population group	0.0%	70.2%	29.8%	100.0%			
			Adjusted residual	-1.6	0.3	0.3				
		Girls	Count	5	46	15	66			
			% within population group	7.6%	69.7%	22.7%	100.0%			
			Adjusted residual	1.9	0.3	-1.1				

	Adolescents	Male	Count	2	17	9	28			
			% within population group	7.1%	60.7%	32.1%	100.0%			
			Adjusted residual	1.0	-0.9	0.6				
		Female	Count	1	44	19	64			
			% within population group	1.6%	68.8%	29.7%	100.0%			
			Adjusted residual	-1.2	0.1	0.4				
	Total		Count	8	140	57	205			
			% within population group	3.9%	68.3%	27.8%	100.0%			
**Food group**	**Population group**	**Sex**	**Statistic**	**Never (%)**	**≤ 2/week (%)**	**≥ 3/week (%)**	**Total (excl. NR, %)**	**Pearson χ^2**	**df**	***p*-value**
**Chicken/ poultry**	Children	Boys	Count	0	15	34	49	12.951	3	0.005
			% within population group	0.0%	30.6%	69.4%	100.0%			
			Adjusted residual		-1.2	1.2				
		Girls	Count	0	23	46	69			
			% within population group	0.0%	33.3%	66.7%	100.0%			
			Adjusted residual		-1.0	1.0				
	Adolescents	Male	Count	0	6	21	27			
			% within population group	0.0%	22.2%	77.8%	100.0%			
			Adjusted residual		-1.8	1.8				
		Female	Count	0	36	29	65			
			% within population group	0.0%	55.4%	44.6%	100.0%			
			Adjusted residual		3.5	-3.5				
	Total		Count	0	80	130	210			
			% within population group	0.0%	38.1%	61.9%	100.0%			
**Food group**	**Population group**	**Sex**	**Statistic**	**Never (%)**	**≤ 2/week (%)**	**≥ 3/week (%)**	**Total (excl. NR, %)**	**Pearson χ^2**	**df**	***p*-value**
**Processed meats (cured meats/sausages)**	Children	Boys	Count	2	24	24	50	14.032	6	0.029
			% within population group	4.0%	48.0%	48.0%	100.0%			
			Adjusted residual	-1.3	0.5	0.2				
		Girls	Count	9	34	26	69			
			% within population group	13.0%	49.3%	37.7%	100.0%			

			Adjusted residual	1.7	0.9	-1.8				
	Adolescents	Male	Count	1	6	21	28			
			% within population group	3.6%	21.4%	75.0%	100.0%			
			Adjusted residual	-1.0	-2.7	3.2				
		Female	Count	6	32	28	66			
			% within population group	9.1%	48.5%	42.4%	100.0%			
			Adjusted residual	0.2	0.7	-0.8				
	Total		Count	18	96	99	213			
			% within population group	8.5%	45.1%	46.5%	100.0%			

**Food group**	**Population group**	**Sex**	**Statistic**	**Never (%)**	**≤ 2/week (%)**	**≥ 3/week (%)**	**Total (excl. NR, %)**	**Pearson χ^2**	**df**	***p*-value**
**Fish**	Children	Boys	Count	2	15	33	50	6.466	6	0.373
			% within population group	4.0%	30.0%	66.0%	100.0%			
			Adjusted residual	-0.2	-1.9	2.0				
		Girls	Count	2	31	37	70			
			% within population group	2.9%	44.3%	52.9%	100.0%			
			Adjusted residual	-0.8	0.5	-0.1				
	Adolescents	Male	Count	2	15	11	28			
			% within population group	7.1%	53.6%	39.3%	100.0%			
			Adjusted residual	0.7	1.3	-1.6				
		Female	Count	4	30	35	69			
			% within population group	5.8%	43.5%	50.7%	100.0%			
			Adjusted residual	0.6	0.3	-0.6				
	Total		Count	10	91	116	217			
			% within population group	4.6%	41.9%	53.5%	100.0%			

**Food group**	**Population group**	**Sex**	**Statistic**	**Never (%)**	**≤ 2/week (%)**	**≥ 3/week (%)**	**Total (excl. NR, %)**	**Pearson χ^2**	**df**	***p*-value**
**Vegetables**	Children	Boys	Count	3	15	31	49	2.441	6	0.875
			% within population group	6.1%	30.6%	63.3%	100.0%			
			Adjusted residual	-0.3	1.2	-1.0				
		Girls	Count	4	16	49	69			

Food group	Population group	Sex	Statistic	Never (%)	≤ 2/week (%)	≥ 3/week (%)	Total (excl. NR, %)	Pearson χ^2	df	*p*-value
			% within population group	5.8%	23.2%	71.0%	100.0%			
			Adjusted residual	-0.5	-0.2	0.4				
	Adolescents	Male	Count	2	5	22	29			
			% within population group	6.9%	17.2%	75.9%	100.0%			
			Adjusted residual	0.0	-0.9	0.9				
		Female	Count	6	16	47	69			
			% within population group	8.7%	23.2%	68.1%	100.0%			
			Adjusted residual	0.7	-0.2	-0.2				
	Total		Count	15	52	149	216			
			% within population group	6.9%	24.1%	69.0%	100.0%			
**Food group**	**Population group**	**Sex**	**Statistic**	**Never (%)**	**≤ 2/week (%)**	**≥ 3/week (%)**	**Total (excl. NR, %)**	**Pearson χ^2**	**df**	***p*-value**
**Rice/pasta**	Children	Boys	Count	0	14	36	50	5.227	6	0.515
			% within population group	0.0%	28.0%	72.0%	100.0%			
			Adjusted residual	-0.6	-1.5	1.5				
		Girls	Count	1	25	44	70			
			% within population group	1.4%	35.7%	62.9%	100.0%			
			Adjusted residual	1.4	-0.2	0				
	Adolescents	Male	Count	0	11	18	29			
			% within population group	0.0%	37.9%	62.1%	100.0%			
			Adjusted residual	-0.4	0.1	-0.1				
		Female	Count	0	29	37	66			
			% within population group	0.0%	43.9%	56.1%	100.0%			
			Adjusted residual	-0.7	1.5	-1.4				
	Total		Count	1	79	135	215			
			% within population group	0.5%	36.7%	62.8%	100.0%			
**Food group**	**Population group**	**Sex**	**Statistic**	**Never (%)**	**≤ 2/week (%)**	**≥ 3/week (%)**	**Total (excl. NR, %)**	**Pearson χ^2**	**df**	***p*-value**
**Legumes**	Children	Boys	Count	0	24	27	51	4.720	6	0.580
			% within population group	0.0%	47.1%	52.9%	100.0%			
			Adjusted residual	-0.8	-0.7	0.9				

		Girls	Count	1	37	30	68			
			% within population group	1.5%	54.4%	44.1%	100.0%			
			Adjusted residual	0.6	0.6	-0.7				
	Adolescents	Male	Count	1	13	15	29			
			% within population group	3.4%	44.8%	51.7%	100.0%			
			Adjusted residual	1.5	-0.8	0.5				
		Female	Count	0	37	30	67			
			% within population group	0.0%	55.2%	44.8%	100.0%			
			Adjusted residual	-1.0	0.7	-0.5				
	Total		Count	2	111	102	215			
			% within population group	0.9%	51.6%	47.4%	100.0%			

**Food group**	**Population group**	**Sex**	**Statistic**	**Never (%)**	**≤ 2/week (%)**	**≥ 3/week (%)**	**Total (excl. NR, %)**	**Pearson χ^2**	**df**	***p*-value**
**Bread**	Children	Boys	Count	1	6	43	50	3.978	6	0.680
			% within population group	2.0%	12.0%	86.0%	100.0%			
			Adjusted residual	-0.4	0.5	-0.2				
		Girls	Count	2	9	60	71			
			% within population group	2.8%	12.7%	84.5%	100.0%			
			Adjusted residual	0.0	0.8	-0.8				
	Adolescents	Male	Count	1	0	27	28			
			% within population group	3.6%	0.0%	96.4%	100.0%			
			Adjusted residual	0.3	-1.9	1.6				
		Female	Count	2	7	58	67			
			% within population group	3.0%	10.4%	86.6%	100.0%			
			Adjusted residual	0.1	0.1	-0.1				
	Total		Count	6	22	188	216			
			% within population group	2.8%	10.2%	87.0%	100.0%			

**Food group**	**Population group**	**Sex**	**Statistic**	**Never (%)**	**≤ 2/week (%)**	**≥ 3/week (%)**	**Total (excl. NR, %)**	**Pearson χ^2**	**df**	***p*-value**
**Fruit**	Children	Boys	Count	1	6	42	49	9.256	6	0.160
			% within population group	2.0%	12.2%	85.7%	100.0%			

Food group	Population group	Sex	Statistic	Never (%)	≤ 2/week (%)	≥ 3/week (%)	Total (excl. NR, %)	Pearson χ^2	df	p-value
			Adjusted residual	-1.0	-0.1	0.6				
		Girls	Count	4	4	62	70			
			% within population group	5.7%	5.7%	88.6%	100.0%			
			Adjusted residual	0.5	-2.1	1.5				
	Adolescents	Male	Count	3	6	20	29			
			% within population group	10.3%	20.7%	69.0%	100.0%			
			Adjusted residual	1.6	1.4	-2.1				
		Female	Count	2	11	55	68			
			% within population group	2.9%	16.2%	80.9%	100.0%			
			Adjusted residual	-0.8	1.1	-0.5				
	Total		Count	10	27	179	216			
			% within population group	4.6%	12.5%	82.9%	100.0%			

Food group	Population group	Sex	Statistic	Never (%)	≤ 2/week (%)	≥ 3/week (%)	Total (excl. NR, %)	Pearson χ^2	df	p-value
**Milk**	Children	Boys	Count	0	0	50	50	13.316	6	0.038
			% within population group	0.0%	0.0%	100.0%	100.0%			
			Adjusted residual	-1.4	-1.1	1.8				
		Girls	Count	1	0	69	70			
			% within population group	1.4%	0.0%	98.6%	100.0%			
			Adjusted residual	-0.8	-1.4	1.5				
	Adolescents	Male	Count	1	0	27	28			
			% within population group	3.6%	0.0%	96.4%	100.0%			
			Adjusted residual	0.3	-0.8	0.3				
		Female	Count	4	4	61	69			
			% within population group	5.8%	5.8%	88.4%	100.0%			
			Adjusted residual	1.9	3.0	-3.4				
	Total		Count	6	4	207	217			
			% within population group	2.8%	1.8%	95.4%	100.0%			

Food group	Population group	Sex	Statistic	Never (%)	≤ 2/week (%)	≥ 3/week (%)	Total (excl. NR, %)	Pearson χ^2	df	p-value
**Yoghurt**	Children	Boys	Count	0	3	46	49	12.065	6	0.061

Food group	Population group	Sex	Statistic	Never (%)	≤ 2/week (%)	≥ 3/week (%)	Total (excl. NR, %)	Pearson χ^2	df	*p*-value
			% within population group	0.0%	6.1%	93.9%	100.0%			
			Adjusted residual	-0.8	-2.0	2.2				
		Girls	Count	0	9	61	70			
			% within population group	0.0%	12.9%	87.1%	100.0%			
			Adjusted residual	-1.0	-0.7	0.9				
	Adolescents	Male	Count	1	4	24	29			
			% within population group	3.4%	13.8%	82.8%	100.0%			
			Adjusted residual	1.5	-0.2	-0.2				
		Female	Count	1	17	50	68			
			% within population group	1.5%	25.0%	73.5%	100.0%			
			Adjusted residual	0.6	2.7	-2.8				
	Total		Count	2	33	181	216			
			% within population group	0.9%	15.3%	83.8%	100.0%			

Food group	Population group	Sex	Statistic	Never (%)	≤ 2/week (%)	≥ 3/week (%)	Total (excl. NR, %)	Pearson χ^2	df	*p*-value
**Eggs**	Children	Boys	Count	0	34	15	49	8.813	6	0.184
			% within population group	0.0%	69.4%	30.6%	100.0%			
			Adjusted residual	-1.2	1.1	-0.7				
		Girls	Count	0	45	25	70			
			% within population group	0.0%	64.3%	35.7%	100.0%			
			Adjusted residual	-1.6	0.3	0.2				
	Adolescents	Male	Count	1	15	13	29			
			% within population group	3.4%	51.7%	44.8%	100.0%			
			Adjusted residual	0.4	-1.3	1.2				
		Female	Count	4	42	22	68			
			% within population group	5.9%	61.8%	32.4%	100.0%			
			Adjusted residual	2.4	-0.2	-0.5				
	Total		Count	5	136	75	216			
			% within population group	2.3%	63.0%	34.7%	100.0%			

Food group	Population group	Sex	Statistic	Never (%)	≤ 2/week (%)	≥ 3/week (%)	Total (excl. NR, %)	Pearson χ^2	df	*p*-value

**Canned foods**	Children	Boys	Count	21	25	1	47	19.370	6	0.004
			% within population group	44.7%	53.2%	2.1%	100.0%			
			Adjusted residual	1.2	-0.6	-1.2				
		Girls	Count	30	37	3	70			
			% within population group	42.9%	52.9%	4.3%	100.0%			
			Adjusted residual	1.2	-0.8	-0.6				
	Adolescents	Male	Count	10	13	6	29			
			% within population group	34.5%	44.8%	20.7%	100.0%			
			Adjusted residual	-0.3	-1.4	3.7				
		Female	Count	17	44	2	63			
			% within population group	27.0%	69.8%	3.2%	100.0%			
			Adjusted residual	-2.0	2.5	-1.0				
	Total		Count	78	119	12	209			
			% within population group	37.3%	56.9%	5.7%	100.0%			

Values are shown as counts, row percentages within each age–sex population group, and adjusted residuals from the Pearson χ^2 test. Intake categories were collapsed as Never, ≤2/week, and ≥3/week. Percentages were calculated among respondents with valid FFQ data for each food item; NR/missing responses were excluded from the denominators. Pearson χ^2 statistics, degrees of freedom, and p-values refer to the overall comparison across the four age–sex groups for each food group. For chicken/poultry, no participants were classified in the Never category in the reanalysis output; this category was retained as 0 for consistency across food groups. The FFQ data were reanalysed from historical age–sex-stratified aggregate frequency tables. Original response categories were regrouped at the aggregate-table level; therefore, valid denominators may differ slightly among food groups and from the corresponding hair-analytical subgroup sizes. These differences reflect the structure of the historical aggregated FFQ outputs and item-specific non-reporting and do not indicate participant duplication.

Table S2. Exploratory contrasts in FFQ consumption frequency across age–sex groups using Pearson χ^2 tests with Benjamini–Hochberg FDR adjustment, by food group.

Food group	Children overall ≥3/week (%, excl. NR)	Adolescents overall ≥3/week (%, excl. NR)	Pearson χ^2 (df)	*p*-value	FDR *q*-value (BH)	*N* valid
Red meat	27.4	29.0	3.966 (6)	0.681	0.733	206
Pork	25.7	30.4	6.999 (6)	0.321	0.562	205
Chicken/poultry	67.8	54.3	12.951 (3)	**0.005**	**0.035**	210
Processed meats (cured meats/sausages)	42.0	52.1	14.032 (6)	**0.029**	0.133	213
Fish	58.3	47.4	6.466 (6)	0.373	0.580	217
Vegetables	67.8	70.4	2.441 (6)	0.875	0.875	216
Rice/pasta	66.7	57.9	5.227 (6)	0.515	0.721	215
Legumes	47.9	46.9	4.720 (6)	0.580	0.733	215
Bread	85.1	89.5	3.978 (6)	0.680	0.733	216
Fruit	87.4	77.3	9.256 (6)	0.160	0.368	216
Milk	99.2	90.7	13.316 (6)	**0.038**	0.133	217
Yoghurt	89.9	76.3	12.065 (6)	**0.061**	0.171	216
Eggs	33.6	36.1	8.813 (6)	0.184	0.368	216
Canned foods	3.4	8.7	19.370 (6)	**0.004**	**0.035**	209

Percentages were recalculated from the updated valid population group × intake crosstabs by pooling boys and girls for children, and male and female adolescents for adolescents. FDR *q*-values were recalculated across the 14 food-group Pearson χ^2 *p*-values using the Benjamini–Hochberg procedure. NR/missing responses were excluded from the denominators because the reanalysis tables were based on valid cases for each food item.

Table S3. Summary of left-censoring percentages (% <LoD) by element, age group, and sex.

Element	Children, all	Boys	Girls	Adolescents, all	Adolescent males	Adolescent females
**B**	89.2	90.0	88.6	94.8	96.6	94.1
**Ca**	46.7	80.0	22.9	2.1	3.4	1.5
**Co**	25.8	38.0	17.1	23.7	37.9	17.6
**Fe**	0	0	0	0	0	0
**Li**	86.7	90.0	84.3	98.97	100	98.5
**Mg**	7.5	14.0	2.9	0	0	0
**Mo**	0	0	0	0	0	0
**Ni**	51.7	76.0	34.3	43.3	72.4	30.9
**Se**	0	0	0	0	0	0

Values indicate the percentage of observations below the analytical limit of detection. For nickel, percentages correspond to the harmonised censoring-aware Ni field used for formal inference. The legacy substitution-based child Ni† field is not included in this summary table. A direct numerical comparison of Ni† and the final harmonised Ni field is provided in Supplementary Table S4.

Table S4. Audit comparison of the legacy substitution-based child nickel field (Ni†) and the final harmonised censoring-aware nickel field (Ni).

Comparison component	Legacy Ni†	Final harmonised Ni	Interpretive consequence
**Analytical population**	Children aged 6–9 years; historical processed field	Children aged 6–9 years; final harmonised field	Same underlying child cohort; only data handling differs.
**Treatment of <LoD observations**	Manual substitution in the historical processing workflow	Explicit left-censoring; NADA/NADA2 workflow, with ROS summaries where supported	The harmonised field preserves censoring information.
**Overall median (IQR), μg g⁻¹**	0.23 (0.16–0.50)	0.060 (0.025–0.168)	Central estimates differed materially.
**P95 (95% CI), μg g⁻¹**	1.04 (0.96–1.12)	0.586 (0.375–1.056)	Upper-tail estimation was also sensitive to nondetect handling.
**P97.5, μg g⁻¹**	Not reported in the historical field	0.866	P97.5 is retained only for the final harmonised distribution.
**Boys: median (IQR), μg g⁻¹**	0.42 (0.22–0.56)	0.00568 (0.00091–0.035)	The harmonised estimate is model-derived under substantial censoring.
**Girls: median (IQR), μg g⁻¹**	0.22 (0.15–0.49)	0.117 (0.054–0.201)	Girls showed higher values in the harmonised distribution.
**Sex comparison**	Not significant (historical analysis)	$p = 2.99 \times 10^{-5}$; BH $q = 7.98 \times 10^{-5}$	Inference changed after explicit censoring was applied.
**Role in the present manuscript**	Transparency and methodological sensitivity only	Primary field for all descriptive, sex, age, zone, correlation, and hair–soil analyses	Ni† is excluded from primary inference.

Ni† reproduces the historical substitution-based summaries previously reported for the child dataset [39]; these values were not re-analysed in the final NADA/NADA2 workflow. Final harmonised Ni values derive from the definitive JoX analysis package and supersede earlier intermediate censoring-aware estimates. The comparison is presented as a methodological sensitivity audit and not as a formal paired method-validation study. Model-derived ROS estimates may fall below the analytical LoD and do not represent directly observed concentrations. Only the harmonised Ni field was used for formal inference and direct comparison with adolescents.

Table S5. Detailed analytical distribution of scalp-hair elements in children aged 6–9 years from Alcalá de Henares, Spain.

Element	*N* total	*n* > LoD	% < LoD	LoD (μg g^{-1})	Mean (μg g^{-1})	Median (IQR) (μg g^{-1})	P95 (95% CI) (μg g^{-1})	P97.5 (μg g^{-1})	Detected range (μg g^{-1})
B	120	13	89.2	2.68	—	Detectability only	—	—	2.73–7.42
Ca	120	64	46.7	162	233	168 (102–244)	509 (405–691)	665	162–706
Co	120	89	25.8	0.00343	0.00941	0.00632 (0.0034–0.011)	0.026 (0.02–0.036)	0.033	0.00357–0.067
Fe	120	120	0	1.81	8.42	7.54 (6–9.3)	12.8 (11.5–23.3)	17.9	2.21–46.8
Li	120	16	86.7	0.00938	—	Detectability only	—	—	0.00947–0.135
Mg	120	111	7.5	2.03	14.2	9.05 (4.85–15.9)	36.6 (31.3–44.6)	41.6	2.2–213
Mo	120	120	0	0.00813	0.034	0.03 (0.022–0.037)	0.067 (0.046–0.078)	0.078	0.01–0.22
Ni	120	58	51.7	0.02	0.168	0.06 (0.025–0.168)	0.586 (0.375–1.06)	0.866	0.047–2.47
Se	120	120	0	0.115	0.343	0.318 (0.269–0.367)	0.486 (0.43–0.528)	0.511	0.157–1.61

N total = number analysed; *n* > LoD = number quantified above the analytical limit of detection. Mean denotes the arithmetic mean for uncensored elements or the reverse-Kaplan–Meier mean for censored elements; censored medians, IQRs, P95 and P97.5 values were estimated using censoring-aware methods where supported. P95 confidence intervals were estimated using a 20,000-replicate nonparametric percentile bootstrap of participant-level records. "Detectability only" indicates that high censoring or sparse detections precluded stable distributional estimation. Detected range excludes censored observations. Model-derived estimates for censored distributions may fall below the analytical LoD and should not be interpreted as directly observed concentrations. Concentrations are expressed as μg g^{-1} and numerical concentrations are presented to a maximum of three significant figures.

Table S6. Detailed analytical distribution of scalp-hair elements in boys aged 6–9 years from Alcalá de Henares, Spain.

Element	*N* total	*n* > LoD	% < LoD	LoD (μg g⁻¹)	Mean (μg g⁻¹)	Median (IQR) (μg g⁻¹)	P95 (95% CI) (μg g⁻¹)	P97.5 (μg g⁻¹)	Detected range (μg g⁻¹)
B	50	5	90	2.68	—	Detectability only	—	—	2.73–7.37
Ca	50	10	80	162	—	Detectability only	—	—	162–337
Co	50	31	38	0.00343	0.00611	0.00474 (0.00306–0.00793)	0.013 (0.01–0.015)	0.014	0.00357–0.016
Fe	50	50	0	1.81	7.88	6.59 (5.23–8.76)	12.2 (10.1–32.7)	14.8	2.21–46.8
Li	50	5	90	0.00938	—	Detectability only	—	—	0.00947–0.135
Mg	50	43	14	2.03	11	5.94 (3.23–9.09)	16.2 (11.3–133)	31.2	2.2–213
Mo	50	50	0	0.00813	0.034	0.031 (0.025–0.036)	0.065 (0.044–0.086)	0.076	0.013–0.093
Ni	50	12	76	0.02	0.14	0.00568 (0.00091–0.035)	0.389 (0.141–1.74)	0.759	0.047–2.47
Se	50	50	0	0.115	0.331	0.321 (0.287–0.371)	0.463 (0.407–0.507)	0.495	0.23–0.511

N total = number analysed; *n* > LoD = number quantified above the analytical limit of detection. Mean denotes the arithmetic mean for uncensored elements or the reverse-Kaplan–Meier mean for censored elements; censored medians, IQRs, P95 and P97.5 values were estimated using censoring-aware methods where supported. P95 confidence intervals were estimated using a 20,000-replicate nonparametric percentile bootstrap of participant-level records. "Detectability only" indicates that high censoring or sparse detections precluded stable distributional estimation. Detected range excludes censored observations. Model-derived estimates for censored distributions may fall below the analytical LoD and should not be interpreted as directly observed concentrations. Concentrations are expressed as μg g^{-1} and numerical concentrations are presented to a maximum of three significant figures.

Table S7. Detailed analytical distribution of scalp-hair elements in girls aged 6–9 years from Alcalá de Henares, Spain.

Element	*N* total	*n* > LoD	% < LoD	LoD (μg g⁻¹)	Mean (μg g⁻¹)	Median (IQR) (μg g⁻¹)	P95 (95% CI) (μg g⁻¹)	P97.5 (μg g⁻¹)	Detected range (μg g⁻¹)
B	70	8	88.6	2.68	—	Detectability only	—	—	3.35–7.42
Ca	70	54	22.9	162	276	222 (165–298)	622 (444–702)	694	163–706
Co	70	58	17.1	0.00343	0.012	0.00755 (0.00465–0.014)	0.03 (0.021–0.044)	0.038	0.00375–0.067
Fe	70	70	0	1.81	8.81	7.86 (6.74–9.6)	13.1 (11.6–23.3)	19.3	3.38–37.9
Li	70	11	84.3	0.00938	—	Detectability only	—	—	0.00975–0.026
Mg	70	68	2.9	2.03	16.5	13.4 (7.96–23.2)	38.4 (32.3–44.6)	42.4	2.65–45.6
Mo	70	70	0	0.00813	0.033	0.029 (0.022–0.038)	0.061 (0.044–0.078)	0.074	0.01–0.22
Ni	70	46	34.3	0.02	0.196	0.117 (0.054–0.201)	0.587 (0.383–1.06)	0.811	0.069–1.25
Se	70	70	0	0.115	0.351	0.316 (0.262–0.365)	0.493 (0.428–1.46)	0.784	0.157–1.61

N total = number analysed; *n* > LoD = number quantified above the analytical limit of detection. Mean denotes the arithmetic mean for uncensored elements or the reverse-Kaplan–Meier mean for censored elements; censored medians, IQRs, P95 and P97.5 values were estimated using censoring-aware methods where supported. P95 confidence intervals were estimated using a 20,000-replicate nonparametric percentile bootstrap of participant-level records. “Detectability only” indicates that high censoring or sparse detections precluded stable distributional estimation. Detected range excludes censored observations. Model-derived estimates for censored distributions may fall below the analytical LoD and should not be interpreted as directly observed concentrations. Concentrations are expressed as μg g⁻¹ and numerical concentrations are presented to a maximum of three significant figures.

Table S8. Detailed analytical distribution of scalp-hair elements in adolescents aged 13–16 years from Alcalá de Henares, Spain.

Element	*N* total	*n* > LoD	% < LoD	LoD (μg g^{-1})	Mean (μg g^{-1})	Median (IQR) (μg g^{-1})	P95 (95% CI) (μg g^{-1})	P97.5 (μg g^{-1})	Detected range (μg g^{-1})
B	97	5	94.8	3.34	—	Detectability only	—	—	3.45–4.51
Ca	97	95	2.1	65.3	489	325 (179–652)	1.29×10^3 (993–1.69×10^3)	1.52×10^3	71.8–2.95×10^3
Co	97	74	23.7	0.00149	0.012	0.00381 (0.00167–0.00802)	0.04 (0.026–0.125)	0.106	0.00164–0.164
Fe	97	97	0	1.15	7.66	5.17 (4.32–6.17)	9.61 (7.64–13.6)	12.4	2.67–210
Li	97	1	99	0.02	—	Detectability only	—	—	0.024–0.024
Mg	97	97	0	2.07	43.4	24 (15.7–51.1)	131 (111–176)	156	7.2–241
Mo	97	97	0	0.00693	0.033	0.023 (0.019–0.029)	0.061 (0.033–0.228)	0.168	0.00821–0.438
Ni	97	55	43.3	0.133	0.661	0.169 (0.047–0.39)	2.68 (0.944–4.43)	3.28	0.134–15.8
Se	97	97	0	0.098	0.42	0.323 (0.279–0.381)	0.468 (0.431–0.866)	0.573	0.155–7.96

N total = number analysed; *n* > LoD = number quantified above the analytical limit of detection. Mean denotes the arithmetic mean for uncensored elements or the reverse-Kaplan–Meier mean for censored elements; censored medians, IQRs, P95 and P97.5 values were estimated using censoring-aware methods where supported. P95 confidence intervals were estimated using a 20,000-replicate nonparametric percentile bootstrap of participant-level records. “Detectability only” indicates that high censoring or sparse detections precluded stable distributional estimation. Detected range excludes censored observations. Model-derived estimates for censored distributions may fall below the analytical LoD and should not be interpreted as directly observed concentrations. Concentrations are expressed as μg g^{-1} and numerical concentrations are presented to a maximum of three significant figures.

Table S9. Detailed analytical distribution of scalp-hair elements in male adolescents aged 13–16 years from Alcalá de Henares, Spain.

Element	*N* total	*n* > LoD	% < LoD	LoD (μg g⁻¹)	Mean (μg g⁻¹)	Median (IQR) (μg g⁻¹)	P95 (95% CI) (μg g⁻¹)	P97.5 (μg g⁻¹)	Detected range (μg g⁻¹)
B	29	1	96.6	3.34	—	Detectability only	—	—	3.77–3.77
Ca	29	28	3.4	65.3	381	168 (140–616)	988 (752–1.33 × 10^3)	1.17 × 10^3	71.8–1.33 × 10^3
Co	29	18	37.9	0.00149	0.004	0.0022 (0.00091–0.00336)	0.00702 (0.00367–0.0394)	0.0175	0.00167–0.039
Fe	29	29	0	1.15	11.5	4.52 (3.84–5.32)	6.07 (5.46–210)	67.4	2.67–210
Li	29	0	100	0.02	—	Detectability only	—	—	ND
Mg	29	29	0	2.07	51.2	16 (12.7–94.4)	152 (113–215)	181	7.2–215
Mo	29	29	0	0.00693	0.031	0.021 (0.018–0.027)	0.044 (0.029–0.252)	0.111	0.00821–0.252
Ni	29	8	72.4	0.133	—	Detectability only	—	—	0.39–8.2
Se	29	29	0	0.098	0.351	0.366 (0.292–0.414)	0.461 (0.432–0.626)	0.515	0.155–0.626

N total = number analysed; *n* > LoD = number quantified above the analytical limit of detection. Mean denotes the arithmetic mean for uncensored elements or the reverse-Kaplan–Meier mean for censored elements; censored medians, IQRs, P95 and P97.5 values were estimated using censoring-aware methods where supported. P95 confidence intervals were estimated using a 20,000-replicate nonparametric percentile bootstrap of participant-level records. "Detectability only" indicates that high censoring or sparse detections precluded stable distributional estimation. Detected range excludes censored observations. Model-derived estimates for censored distributions may fall below the analytical LoD and should not be interpreted as directly observed concentrations. Concentrations are expressed as μg g^{-1} and numerical concentrations are presented to a maximum of three significant figures.

Table S10. Detailed analytical distribution of scalp-hair elements in female adolescents aged 13–16 years from Alcalá de Henares, Spain.

Element	N total	n > LoD	% < LoD	LoD (μg g⁻¹)	Mean (μg g⁻¹)	Median (IQR) (μg g⁻¹)	P95 (95% CI) (μg g⁻¹)	P97.5 (μg g⁻¹)	Detected range (μg g⁻¹)
B	68	4	94.1	3.34	—	Detectability only	—	—	3.45–4.51
Ca	68	67	1.5	65.3	536	375 (247–659)	1.29×10^3 (996–1.88×10^3)	1.72×10^3	79.1–2.95×10^3
Co	68	56	17.6	0.00149	0.015	0.00528 (0.00296–0.012)	0.068 (0.026–0.134)	0.126	0.00164–0.164
Fe	68	68	0	1.15	6.01	5.6 (4.71–6.95)	10 (7.94–13.3)	11.9	3.17–14.7
Li	68	1	98.5	0.02	—	Detectability only	—	—	0.024–0.024
Mg	68	68	0	2.07	40.1	24.6 (18.5–49.3)	119 (67.3–140)	135	10.2–241
Mo	68	68	0	0.00693	0.034	0.025 (0.019–0.029)	0.066 (0.034–0.222)	0.131	0.00999–0.438
Ni	68	47	30.9	0.133	0.659	0.226 (0.077–0.378)	2.54 (0.747–2.97)	2.92	0.134–15.8
Se	68	68	0	0.098	0.45	0.319 (0.273–0.362)	0.457 (0.391–1.83)	0.927	0.155–7.96

N total = number analysed; *n* > LoD = number quantified above the analytical limit of detection. Mean denotes the arithmetic mean for uncensored elements or the reverse-Kaplan–Meier mean for censored elements; censored medians, IQRs, P95 and P97.5 values were estimated using censoring-aware methods where supported. P95 confidence intervals were estimated using a 20,000-replicate nonparametric percentile bootstrap of participant-level records. “Detectability only” indicates that high censoring or sparse detections precluded stable distributional estimation. Detected range excludes censored observations. Model-derived estimates for censored distributions may fall below the analytical LoD and should not be interpreted as directly observed concentrations. Concentrations are expressed as μg g^{-1} and numerical concentrations are presented to a maximum of three significant figures.

Table S11. Detailed residential-zone summaries and overall comparisons of scalp-hair element distributions in children.

Element	Zone	*N*	*n* > LoD	% < LoD	Median (IQR) or detectability (μg g⁻¹)	Overall *p*	BH *q*
B	Zone I	24	6	75	6/24 detected	—	—
	Zone II	60	4	93.3	4/60 detected		
	Zone IV	36	3	91.7	3/36 detected		
Ca	Zone I	24	13	45.8	197 (132–249)	0.867	0.902
	Zone II	60	34	43.3	176 (105–238)		
	Zone IV	36	17	52.8	137 (76.2–265)		
Co	Zone I	24	16	33.3	0.00568 (0.00286–0.013)	0.902	0.902
	Zone II	60	46	23.3	0.00662 (0.00362–0.011)		
	Zone IV	36	27	25	0.00676 (0.00392–0.01)		
Fe	Zone I	24	24	0	6.81 (6.13–8.22)	0.134	0.371
	Zone II	60	60	0	7.33 (5.2–9.61)		
	Zone IV	36	36	0	8.26 (7.08–9.25)		
Li	Zone I	24	6	75	6/24 detected	—	—
	Zone II	60	5	91.7	5/60 detected		
	Zone IV	36	5	86.1	5/36 detected		
Mg	Zone I	24	20	16.7	15.8 (4.63–31.5)	0.208	0.416
	Zone II	60	57	5	8.88 (5.95–13.3)		
	Zone IV	36	34	5.6	7.56 (4.84–14.5)		
Mo	Zone I	24	24	0	0.028 (0.021–0.035)	0.328	0.524
	Zone II	60	60	0	0.029 (0.023–0.038)		
	Zone IV	36	36	0	0.032 (0.024–0.037)		
Ni	Zone I	24	8	66.7	8/24 detected	0.57	0.76
	Zone II	60	31	48.3	0.076 (0.035–0.205)		
	Zone IV	36	19	47.2	0.052 (0.031–0.124)		
Se	Zone I	24	24	0	0.276 (0.255–0.336)	0.008	0.064
	Zone II	60	60	0	0.318 (0.268–0.36)		
	Zone IV	36	36	0	0.341 (0.299–0.424)		

Children were represented in Zones I, II and IV. Values are median (IQR) where stable; otherwise the number detected/number analysed is shown. Censored overall comparisons used NADA::cendiff and uncensored comparisons used Kruskal–Wallis tests. Raw p-values were adjusted across elements using the Benjamini–Hochberg false-discovery-rate procedure. No child-zone comparison remained FDR-significant. B and Li were not subjected to primary zone inference because of high censoring or sparse detections. Concentrations are expressed as μg g^{-1} and numerical concentrations are presented to a maximum of three significant figures.

Table S12. Detailed residential-zone summaries and overall comparisons of scalp-hair element distributions in adolescents.

Element	Zone	*N*	*n* > LoD	% < LoD	Median (IQR) or detectability (μg g^{-1})	Overall *p*	BH *q*
B	Zone I	36	3	91.7	3/36 detected	—	—
	Zone II	20	0	100	0/20 detected		
	Zone III	24	1	95.8	1/24 detected		
	Zone IV	17	1	94.1	1/17 detected		
Ca	Zone I	36	35	2.8	232 (144–290) a	3.44×10^{-13}	2.75×10^{-12}
	Zone II	20	19	5	267 (160–596) ab		
	Zone III	24	24	0	385 (271–545) b		
	Zone IV	17	17	0	967 (768–1.24×10^3) c		
Co	Zone I	36	20	44.4	0.00171 (0.00042–0.00515) a	0.0055	0.0111
	Zone II	20	17	15	0.0046 (0.00294–0.00718) b		
	Zone III	24	21	12.5	0.00491 (0.00308–0.00934) b		
	Zone IV	17	16	5.9	0.00807 (0.00304–0.014) b		
Fe	Zone I	36	36	0	4.83 (4.05–5.83)	0.145	0.185
	Zone II	20	20	0	5.1 (4.69–5.82)		
	Zone III	24	24	0	5.34 (4.25–6.28)		
	Zone IV	17	17	0	6.17 (4.54–8.14)		
Li	Zone I	36	0	100	0/36 detected	—	—
	Zone II	20	0	100	0/20 detected		
	Zone III	24	0	100	0/24 detected		
	Zone IV	17	1	94.1	1/17 detected		
Mg	Zone I	36	36	0	18.8 (14.2–28.8) a	5.65×10^{-7}	2.26×10^{-6}
	Zone II	20	20	0	19.1 (13.8–31.9) a		
	Zone III	24	24	0	24.3 (18.9–35.1) a		
	Zone IV	17	17	0	113 (94.4–131) b		

Element	Zone	*N*	*n* > LoD	% < LoD	Median (IQR) or detectability (μg g⁻¹)	Overall *p*	BH *q*
Mo	Zone I	36	36	0	0.026 (0.019–0.033) a	0.004	0.0106
	Zone II	20	20	0	0.024 (0.021–0.029) a		
	Zone III	24	24	0	0.019 (0.016–0.024) b		
	Zone IV	17	17	0	0.023 (0.02–0.029) ab		
Ni	Zone I	36	20	44.4	0.194 (0.031–0.552)	0.627	0.627
	Zone II	20	9	55	9/20 detected		
	Zone III	24	16	33.3	0.162 (0.058–0.285)		
	Zone IV	17	10	41.2	0.267 (0.116–0.39)		
Se	Zone I	36	36	0	0.304 (0.236–0.369) a	0.0226	0.0361
	Zone II	20	20	0	0.321 (0.289–0.369) ab		
	Zone III	24	24	0	0.321 (0.28–0.353) ab		
	Zone IV	17	17	0	0.378 (0.344–0.399) b		

Adolescents were represented in Zones I–IV. Values are median (IQR) where stable; otherwise the number detected/number analysed is shown. Censored overall comparisons used NADA::cendiff and uncensored comparisons used Kruskal–Wallis tests. Within each element, zones sharing a lowercase letter did not differ after pairwise Benjamini–Hochberg adjustment; letters are shown only for elements whose overall test remained FDR-significant. B and Li were not subjected to primary zone inference because of high censoring. Concentrations are expressed as μg g^{-1} and numerical concentrations are presented to a maximum of three significant figures.
